# The Unique *Liber Sermonum* of Martin of León (c.1130–1203): The Third Crusade, Popular Preaching and the Liturgical Front

**DOI:** 10.1080/03044181.2025.2507801

**Published:** 2025-06-07

**Authors:** Alexander Marx

**Affiliations:** Institute for Medieval Research, Austrian Academy of Sciences, Vienna, Austria

**Keywords:** Third Crusade, crusade preaching, Martin of León, university of Paris, heretics, liturgy, Iberian Peninsula, sermon texts

## Abstract

This article explores the exceptional *Liber Sermonum* composed by Martin of León around 1200. First, it describes the work in its manuscript form, illuminating the historical context of its production. Then, the article maps the occurrence of motifs pertinent to crusading throughout the *Liber*. This data unveils Martin’s expertise and, thus, involvement in several crusade arenas, straddling action against scholastics, Jews, heretics and ‘pagans’ (that is, Muslims). Lastly, three texts of the *Liber* are analysed in-depth: A Palm Sunday sermon evidences Martin’s involvement in depth the Third Crusade and anti-heretical action; a treatise on the Acts of the Apostles shows how he tried to promote popular preaching in León; and a treatise on rogation liturgy elucidates how a providential logic entangled various crusade arenas despite their geographical distance. The methodological tools developed for identifying ‘the crusade’ in these texts demonstrate the pertinence of liturgical sermons for studying the crusade movement.

This article is devoted to the *Liber Sermonum* of the Augustinian canon Martin of León (c.1130–1203), a work of unique nature and composition, which has received little meaningful scholarly attention to date.^1^ It survives in a single copy from San Isidoro in León (MS 11), the basis for the version published in the *Patrologia Latina*. The edition also includes two smaller sermon collections (entitled there *De sanctis* and *De diversis*) as well as four biblical commentaries, on John’s Revelation and the letters James, 1 Peter, and 1 John; in the manuscript, however, all these works are placed in-between the sermons of the *Liber*, to make up a work of roughly 700 folios.

The abbey of San Isidoro had a close relationship with the kings of León and housed their tombs in an impressive Romanesque setting; it was also renowned for holding the remains of Isidore of Seville (c.560–636).^2^ Martin joined this community close to the end of his life, around the time when its scriptorium composed the codex (dated to c.1200); this suggests that he was involved in its production. We also know that Martin was on good terms with Alfonso IX, the king of León (r.1188–1230), serving him as a preacher and confessor; it seems that the young king considered him as an important mentor. Even more significantly, the Leonese queen Berengaria of Castile (r.1197–1204) financed the expensive production of the *Liber*, which was thus composed between 1197 and Martin’s death in 1203. This sponsorship suggests that the work not only served a purpose within the abbey’s walls, but that the court had interests that were related to or could be furthered by these texts. This builds on the assumption that specific goals always underpinned a manuscript’s production; as Richard and Mary Rouse have argued, a codex represents archaeological evidence for the efforts proposed therein.^3^ Since the *Liber* presents itself as a preaching manual, it is worth considering how the royal interests may have dovetailed with San Isidoro’s mission: its regular canons were usually ordained priests and preaching was one of their duties. This article attempts to carve out what these interests were, how these intersected with broader concerns of the Latin West at the time and how Martin’s texts – and hence his activities – in many ways anticipated phenomena that would only be fully developed a generation later.

While the *Liber* tackles a broad range of subjects, straddling registers such as exegesis and liturgy, this article’s vantage point is informed by Martin’s involvement in the crusade movement. This phenomenon permeates his work in meaningful and multifarious ways, but this has not found any acknowledgment in previous scholarship. As the appendix shows, crusade-related matters are a dominant feature of this work. These are defined as elements that played a role in crusade spirituality and thus drove these religious endeavours. By presenting the *Liber* in a way that follows the order of texts in the manuscript, the appendix elucidates its composition; and by exploiting the digitised versions of the *Patrologia Latina*, it catalogues elements that occur therein repeatedly.^4^ However, we run here into a key methodological issue, as terms, such as ‘Jerusalem’, that I identify as ‘crusade elements’, are found in texts that preceded the crusade movement, just as they existed with different layers of meaning in the age of the crusades, as, for example, when a monastery was identified as ‘Jerusalem’. Similarly, militant imagery may refer not only to campaigns against the so-called Infidel, but also to the spiritual warfare, particularly of monks, against vices and demons.^5^ On the other hand, we are facing the issue that medieval Latin did not have a crusade-specific terminology.^6^ We cannot simply identify ‘crusade sermons’, for the genre did not exist as such. It will be a key concern of the following pages to address these methodological challenges. In doing so, I build upon my earlier work developing tools for relating sermon texts broadly to the Third Crusade (1187–92).^7^ As this article will argue, this expedition was also at the epicentre of Martin’s activities.

Martin’s elaborate work can shed new light on crusade spirituality and its diverse components, elucidating in turn Iberia’s nascent involvement in these matters, including the ways in which efforts within Iberia itself intersected with those in the Holy Land. The work also contributes to a better understanding of how crusade mobilisation operated in the late twelfth century, and how Martin tried to promote these efforts by creating his idiosyncratic opus. Essentially anticipating the advent of the Friars, the *Liber* brings protagonists and activities to light which remain invisible in the chronicles that are still the prime sources of crusade scholarship. We do not have a chronicle mentioning Martin’s crusade preaching, but to expect such would be a grave mistake. The chronicles generally broach preaching activities only in passing and in connection to important political figures. Significantly, most authors of the surviving sermon texts remain invisible in these reports, even the most skilled preachers of the period among them, such as Peter Comestor (c.1100–78) or Alan of Lille (c.1120–1203). However, Martin’s own approximately 700 folios are vivid proof that he was a devoted and experienced preacher for the crusade. The *Liber* may not tell us exactly when and where he preached, but it can offer us a great number of other insights. Integrating evidence such as Martin’s *Liber* into the overall picture allows such sources to modify the stories that scholars have hitherto told on the basis of the chronicles.^8^

Moreover, an examination of Martin’s opus will help to locate him within the intellectual context of the early university of Paris and its reform movement around Peter the Chanter (c.1130–97). This network consisted of Paris masters, Victorines, and Cistercians, pursuing the goal of morally reforming Christian society and eager to improve the parish clergy’s abilities for popular preaching. As Jessalynn Bird has aptly shown, this went hand in hand with anti-heretical action and crusading to the East. Contemporaries considered these as entangled arenas, since sin was a collective issue that had to be met on several frontiers.^9^ Martin’s work, which is framed throughout as a preaching manual, offers important but hitherto overlooked evidence for the desire to reach broad audiences via preaching in a period when these efforts were evolving and expanding: the late twelfth century thus prepared the ground for what the Friars would institutionalise a generation later.^10^

This article advances its argument in three steps:

1. The first part describes the *Liber Sermonum* as it appears in the manuscript, to advance the critical argument that the work represents sermon material, including but not limited to the crusade effort. This comprises an analysis of its prologue as well as a consideration of Martin’s *Vita*, which corroborates the interests and ideas present in the *Liber*.
2. The second part is devoted to mapping the multiple crusade arenas in which Martin was involved. These surveys track the programme announced in the *Liber’s* prologue throughout the work. They also permit us to anchor the texts in contemporary phenomena and events from the Iberian Peninsula to the Holy Land.
3. The third part subjects three texts to a close analysis in order to unearth the significance and implications of why crusade motifs appear throughout the *Liber*. A sermon for Palm Sunday (*Sermo 18*) represents a reaction to Jerusalem’s loss in 1187; it evidences Martin’s involvement in the Third Crusade, but also his ongoing (mainly anti-heretical) efforts after this expedition had failed. A treatise devoted to rogation liturgy (*Sermo 29*) sheds light on the providential entanglement of different crusade theatres, often surprisingly conflating distant areas, since these served a common goal. Finally, a piece on the Acts of the Apostles (*De diversis, Sermo 11*) can be described as an elaborate instruction for popular preaching, addressed to the canons of San Isidoro, an effort that strongly blends with promoting crusading in its various forms. This text testifies that Martin belonged to the network of the university of Paris, I argue, and it elucidates the *Liber’s* purpose as a rich repository meant for preaching beyond the abbey.

## The *Liber Sermonum*: Biographical Context, Manuscript and Purpose

The perception of Martin was hitherto largely informed by the *Vita Sancti Martini*, penned by Lucas of Tuy (d.1249), likely in the 1220s. This text anchors Martin in León, the place where his life began and ended, in an apparently unsuccessful attempt at canonising him.^11^ The connection to Léon is reinforced by the location of the *Liber’s* only surviving copy and an entry in the abbey’s obituary that notes his death in 1203. However, the *Liber* itself undermines this connection, strongly suggesting that he was a Paris-trained theologian: it recycles numerous Parisian works, in particular those of Peter Lombard (c.1100–60) and the *Glossa Ordinaria*, the authoritative exegetical landmark of the period. It is quite clear that these works were not available in the Peninsula: if not in Paris, Martin must have studied them elsewhere.^12^ The fact that he used the *Glossa* for multiple books of the Bible makes apparent that he had a well-stocked library at his disposal, which certainly surpassed the capacities of San Isidoro (the enormous *Glossa* would usually be spread over several codices).^13^ A possible stay in Paris is supported by a plausible chronological argument: born around 1130, Martin would have studied there in the 1150s, the heyday of Peter Lombard and his circle.^14^ The *Vita* encourages this supposition by noting that he was educated by ‘a master’ (*docebatur a magistro*) (PL 208: 12). The *Liber’s* prologue, which will be examined below, admits already that the work draws heavily on preexisting materials.^15^ Indeed, on numerous occasions, entire paragraphs are taken from other works such as the *Glossa* or those of Gregory the Great (c.540–604) – but more often than not the sources remain undisclosed. However, the prologue overstates Martin’s direct reliance on others’ writing: the *Liber* contains many texts that apparently stem from his own pen (running them through the full text databases does not yield any other attributions). Similarly, analysing his use of sources reveals that he also freely adapted them to his needs, often updating the authority in light of contemporary phenomena.^16^

Even though the nature and sources of the *Liber* strongly indicate that Martin was based for some time in northern France, we cannot say much about the stages of his life. It is a curious fact that the *Vita* omits roughly 40 years from its account. After an introduction and three chapters on his childhood in León (chs.1–4; PL 208: 9–13), it jumps to a pilgrimage that Martin made to Rome, explicitly dated to the pontificate of Urban III (Nov. 1185 to Oct. 1187) – thus leaping without any words of explanation or transition from Martin’s adolescence, probably in the 1140s, to the mid-1180s. After his sojourn in Italy, he travelled to the Holy Land (*Jerosolymam adiit*), where he stayed for two years, which means that he was in the East around the time of the Third Crusade. There he served the Knights Hospitaller (*sancto Jerosolymitano hospitali tanta humilitate ac gratia praeditus biennio deservivit*) (chs.5–6; PL 208: 13). This plausibly relates to crusading activities, and his presence at the time suggests that he may even have been a participant of the Third Crusade.^17^ Eventually, Martin returned to León, but with stopovers in Constantinople, Paris, Tours, Canterbury, Ireland, and southern France. This clearly indicates that he actually travelled extensively in his lifetime (chs.7–8; PL 208: 14). It was only then that he joined the canons of San Isidoro; he had been a secular cleric before (*permanente in habitu saeculari*), so notes the *Vita* (PL 208: 12).^18^

The remainder of the *Vita* is devoted to Martin’s last ten years or so in León, clearly with the goal of anchoring him there (chs.9–24; PL 208: 15–24). Indeed, the *Vita’s* first chapter had already done so by relating how Isidore of Seville appeared before Martin and granted him the wisdom that guided him henceforth in his profound understanding of the Bible (PL 208: 11).^19^ Whereas his exegetical training certainly had been sought elsewhere, the *Vita* prefers to attribute it to divine inspiration, entangling a foreign Martin with León’s local hero. The suspicious gap of forty years suggests that he spent a significant portion of his life beyond León and that Lucas purposefully omitted these parts, since they would have hampered the goal of granting him a specifically Leonese identity; but neither do we have much data to document Martin’s presence and activities in the Peninsula.^20^ In conclusion, comparing the *Vita* and the *Liber* reveals two different figures. These observations agree with Patrick Henriet’s argument that Lucas was in general someone who adapted matters freely to his needs. Significantly, he also tried to appropriate other figures – including Aristotle – for León.^21^ Nevertheless, the *Vita* also offers valuable hints about Martin’s activities, and mistrusting these details does not seem fruitful, since such hagiographic texts were bound to plausibility and their audience’s horizon of knowledge.^22^ Like Martin, Lucas was a canon in San Isidoro, and writing only some years after Martin’s death; and as I will argue, the details of the *Vita* are confirmed by the interests and expertise Martin displays in the *Liber* itself.

The *Vita* offers unique evidence on the *Liber’s* genesis: Having returned from the Holy Land, Martin commissioned the production of the surviving Leonese codex. He dictated his extant texts to those working in the abbey’s scriptorium (*tradebat scriptoribus, qui ab eo dictata vel copillata scribebant, transferentes in pergamena*) (PL 208: 16). A team of scribes operated under his supervision: the *Liber* seems to have been a collective project. The story brings to the fore that Martin already brought his texts in some form to León, likely unsuitable for preservation, perhaps worn by avid use – and they possibly had not yet taken the form in which they are arranged in the extant codex. The *Vita’s* same chapter outlines that Alfonso IX and his noblemen frequently listened to Martin’s sermons. The juxtaposition of these two themes strongly suggests that the work served as the main source for his preaching.^23^ Amélie de las Heras has underlined that his texts had such a purpose; some of them even resemble the *sermo modernus*, the new type of sermon developed in contemporary Paris.^24^ The *Vita* also emphasises that the *Liber’s* erudition outmatches that of other theological masters (*quosque magistros theologos superaret*) and that Martin should be counted among the great Church Fathers. Lucas likewise highlights the *Liber’s* purpose in combatting Jews and heretics (*Judaeorum perfidia confutatur, singulatim omnes haereses expugnantur*), thus pointing to crusading activities and other matters beyond the abbey’s walls (PL 208: 11 and 17).

Judging from both the *Vita* and the extant codex, then, the work as it stands today was composed in León in the last years of Martin’s life, but drawing on preexisting texts. These conclusions lead us to two research agendas: (a) to consider what Martin, the other canons and the royal court intended to achieve with the final version; and (b) to investigate specific texts that were reproduced on this occasion, but that stemmed from earlier efforts. These texts allow us to trace Martin’s earlier activities. They possibly underwent adaptations of purpose in-between their original composition and their manifestation in the current form, but we must not overstate the matter.^25^ Martin’s original sermons may have had a different form, yet there is not any sound reason to suppose that the intellectual content would have been substantially different. The more often ideas occur in the *Liber* the more likely it is that these had actually been used in preaching; successfully tested, they were thus selected for the *Liber*.

The manuscript of the *Liber* consists of two codices set up as an ensemble: both have the same format and are written in the same set of hands.^26^ They are large, at 49 × 34 cm, and in a clean state – much different from typical sermon codices, such as those surviving from Paris, which are often small and incomplete. Apart from format, however, one finds in the *Liber* many devices known from other sermon manuscripts. This includes a table of contents at the front of both volumes, devoted to recording each text’s title and number (in both entitled: *capitula in libro sermonum*). It also includes running titles at the top of each page, consistently applied throughout the two codices, a perfect device for navigating this vast work. Moreover, there are numerous pointers (often figure-shaped arrows) that highlight specific passages, and glosses that mostly note an authority cited in the main text – Peter Lombard or Augustine, for example. All these organising devices were designed for enabling a recipient to effectively exploit the material for the purposes of preaching. Martin himself announces this principle in the work’s preface, a short text that precedes the prologue in both codices: he aims at finding what one searches even easier (*quod quaeritur facilius occurrat*). This is a verbatim quotation from Peter Lombard’s *Sententiae*. The *Liber’s* organisation demonstrates that Martin had internalised these innovative approaches, after having been trained in the same, perhaps by the Lombard himself.^27^ Besides the two codices, another division is found within the first codex: a new foliation starts with *Sermo 5* (On the Epiphany), yet this does not herald the start of another work. The same is true throughout the entire two volumes; nowhere do we find any paratext to mark the incipit of another work or introduction of another author: the manuscript presents its roughly 700 folios as one opus magnum. Henceforth, it will be cited accordingly: MS 11/1, the first foliation in the first volume; MS 11/2, the second foliation; and MS 11/3, the second volume.

The fact that the Leonese queen financed this lavish manuscript indicates that specific interests in León facilitated its production.^28^ It is highly unlikely, however, that these were related to the attempt to canonise Martin, given that the Martins of the *Liber* and *Vita* diverge so significantly, and that there was a whole generation in-between the composition of the two texts. Similarly, one would expect a more easily manageable work, which the curia or other stakeholders could easily review. It is likewise highly unlikely that the Leonese interest consisted merely in preserving Martin’s texts: the *Liber’s* prologue clearly betrays another impetus, as we shall see. Moreover, the manuscript does not show any explicit attribution to Martin. In either case – the hagiographical or preservation-driven origin – one would expect a clearly marked attribution. Yet, the manuscript displays all signs of a complete production (all titles and embellishing features are there), which we can thus consider as purposeful. The anonymous form ties in nicely with the *Vita’s* assertion that Martin was the driving force behind this opus, but that the codex originated as a collective project of the canons.

The *Liber’s* anonymous preservation is very typical for sermon material; care was not so much about the original author as about a text’s usefulness for preaching.^29^ The work’s paratextual framings identify it clearly as preaching material. This is consistent throughout the two codices, starting with its title of *Liber Sermonum*, which is repeated on several occasions. The *Liber* is also coherently organised according to the liturgical calendar: With the exception of twelve texts with non-liturgical titles, each text is entitled as a *sermo* and ascribed to a specific feast, both in the table of contents and at the texts themselves, for a total of 54 texts ascribed to 30 different feasts. The paratexts unmistakably tell us that this is a sermon collection, that is, a resource for preaching activities, and a good number of the individual texts follow sermon form in rhetoric and structure.^30^ However, a notable disparity reveals itself here: as this essay’s appendix shows, while some comply well with a typical sermon’s length, others are of a remarkable size. Many texts come in at around 20 folios, and an exceptional piece at almost 200 folios, which still bears the title of *Sermo quartus in natale domini*, a sermon for Christmas (fols. 20v–212v, henceforth *Sermo 4*). The edition divides this item into 38 sections (PL 208: 83–549), though these divisions do not exist in the manuscript. This curious finding raises questions of nature and purpose as well as of why the longer texts of the *Liber* were even entitled as sermons. Yet, one cannot ignore the fact that someone, probably Martin himself, believed this to be the appropriate label. Two of these enigmatic texts will be analysed in the case studies below.

All these observations contest past characterisations of the work. One of these was based on its own preface, which speaks of a *Concordia* of Old and New Testament (PL 208: 27). This label is also found in the *Vita* (*duo nimiae magnitudinis volumina edidit, quae Concordia nominantur*) when addressing the *Liber’s* composition and purpose, as already discussed above (PL 208: 11). The fulfilment between Old and New Testament is an important trait of Martin’s work; but as this article’s appendix demonstrates, so are many other traits. Thus, if it is useful at all, *Concordia* may only apply to parts of the *Liber*, just as it may more sketch a method than denote a genre. Adeline Rucquoi and Antonio Viñayo González, on the other hand, have spoken of the text as an anti-Jewish treatise.^31^ As will be discussed below, this applies only to one part. Such misleading characterisations are symptomatic of previous discussions of the *Liber* that have remained unsystematic. Nor have scholars considered how historical phenomena may have granted the *Liber* shape and purpose, understanding it instead as an exclusively monastic or exegetical effort. This points to a broader issue in sermon studies, where the interpretation of such sources is often limited in similar fashion.^32^

The *Liber’s* prologue substantiates its dedication to preaching, but also its engagement with historical phenomena (PL 208: 29–32). An identical text is placed at the beginning of both codices; this makes apparent that a plan was underlying the work’s composition (MS 11/1, fols. 1r–2r and MS 11/3, fols. 1v–2v). As is visible in the appendix, the prologue announces a programme that is realised throughout the work. It addresses Martin’s brothers in San Isidoro: the work’s final version was thus meant for local implementation. It also notes that he started composing it in 1185. Given its length, he must have worked on it for some years, including the time of the Third Crusade. Bringing this fact together with the *Vita’s* report, he seems to have started it while being an itinerant preacher before realising the final form in the scriptorium of San Isidoro.^33^ This would be typical for sermon material, which always stood in reciprocity to its delivery. Henry of Albano (c.1135–89), for example, penned his opus while preaching the Third Crusade.^34^ In the prologue, Martin calls his brothers to practice both *lectio* and *praedicatio*, drawing on the three-fold scheme of Peter the Chanter, where *praedicatio* designated popular preaching, though omitting *disputatio*, a clue that Martin’s work did not address intellectual debates.^35^ He calls the canons to teach others by word and example, that is, to preach (*aliis verbo et exemplo viam rectam ostendunt*).^36^ Importantly, the *Liber* shows them how to do that (*ibi invenietis qualiter vos oporteat vivere et alios docere*) (PL 208: 32). Martin presents this as a handbook for preachers; promoting the Parisian agenda, he likely hoped to entice the canons to preach regularly beyond the cloister. This generally complies with why sermon collections were penned in the period, just as it mirrors the efforts of other Parisian theologians such as Thomas of Chobham (c.1160–c.1230), who realised this agenda in the diocese of Salisbury.^37^ The prologue also outlines the material’s objectives, including that preaching prepares for the Last Judgement.^38^ This is very much indebted to the idea that sin was a collective issue; and it significantly incorporates crusading, since the Apocalypse was expected in Jerusalem.^39^ Consequently, the prologue calls for fortifying the Catholic Church’s faith (*catholicae Ecclesiae fides roboratur*), which is a key concept of the *Liber*: the word robor* appears 101 times in the text. It then proceeds to enumerate the groups against which the material should be used: philosophers, Jews, heretics and pagans, that is, Muslims (PL 208: 31–32). It thus announces crusade preaching as a vital purpose of this enormous work.

Since Martin refers to *lectio* in the prologue, it remains possible that the multifaceted *Liber* also had a purpose within San Isidoro (a subject that I leave to other scholars). However, it has become clear that the work was certainly entangled with efforts beyond its walls. This is not surprising: the regular canons were usually ordained priests and thus required to preach. Thanks to effectively cooperating with both the king and the bishop of León, San Isidoro supervised an extensive parish network. It even strengthened its control over the parishes in this period, as attested by multiple references in local sources that the canons frequently visited the parishes and were sometimes even responsible for administering them.^40^ Thus, Martin did not compose this work in a vacuum, or with illusionary goals in mind, but there was an ecclesiastical structure that made the endeavour into a promising one. The *Liber* may even have been answering a demand.^41^ While the *Vita* reports that Martin preached to León’s elites, the parish network suggests that his texts may have been intended for even broader audiences. It is now time to consider how this may have intersected with crusade efforts, since recent research has made clear that such were also very much a matter of local initiatives.^42^

## Mapping Martin’s Multiple Crusade Frontiers

The enemy-related spectrum announced in the *Liber’s* prologue straddles heretics and pagans as well as philosophers and Jews – the latter two were often collateral damage of crusade efforts.^43^ These groups relate to diverse historical arenas: the Muslims to both the Iberian Peninsula and the Holy Land; heretical groups especially but not limited to southern France; and the philosophers to the northern French schools. As we have seen, all these places are present in the *Vita* as stations in Martin’s life: this is certainly not a coincidence. The spectrum points to a number of significant phenomena: (1) The Iberian Peninsula witnessed constantly shifting political and religious borders at the time; it remained in an undecided equilibrium until the Christian victory of Las Navas de Tolosa (1212).^44^ (2) The crusade movement gained new force after both the relic of the True Cross and Jerusalem had been lost in 1187: these events transformed the concept of crusading and the nature of crusading calls.^45^ (3) Anti-heretical efforts increased significantly, eventually fusing into the Albigensian Crusade (1209–29) and the establishment of inquisitorial procedures.^46^ (4) The early university of Paris developed into an eminent centre of learning, but it also hosted different groups with divergent interests and approaches.^47^ Martin’s work thus addresses several key developments of the period and can elucidate how all these phenomena were entangled, sometimes in surprising ways.

Surveying the *Liber* reveals Martin’s considerable interest in all four of these clusters, thus bringing his expertise and experience to the fore. If the interests he demonstrates can be read as an indication of the contexts in which he was active, his presence at a specific time and place is still difficult to prove. It is important to consider as well the possibility that what might seem a pertinent object was not directly related to crusading; as is the case with the ‘hermeneutical Jew’ about whom Jeremy Cohen has written, it may remain a textual or exegetical phenomenon, rather than referring to actual groups or places.^48^ However, it is unwarranted to assume that clerics would have operated in a vacuum. We know of many authors who were engaged in various historical actions, including many whose monastic ideal would have demanded otherwise. As John Baldwin, Philippe Buc, and Jessalynn Bird have shown, this is especially true for the reform movement, which had the explicit goal of engaging with society via preaching and pastoral care, while agitating against those theologians who did not do so.^49^ In particular Katherine Allen Smith and Cecilia Gaposchkin have unearthed a strong tendency since the time of the First Crusade (1095–99), to intrinsically integrate crusading into the exegetical and liturgical register, since it was such a pivotal concern in contemporary society. Gaposchkin has even argued that the Jerusalem of the liturgy always comprised the actual earthly city in this historical period: the different senses of Scripture (literal, anagogical, allegorical, and moral) were indistinguishable – not antithetical. This was due to broader exegetical developments that granted a previously unknown prominence to the literal sense, which can be identified with the earthly Jerusalem and hence the crusade.^50^

Within this historical context, and given Martin’s educational background, the appearance of pertinent elements in his work strongly lends itself to the crusading purpose. As discussed in the prologue, the *Liber* is dedicated to providing resources for agitating against certain groups, who are thus a target for both preaching and crusading. Martin answers therein the vital question of who was aligned with the forces of evil and who, consequently, poses a threat to the Christian community.^51^ Cohen himself has argued that an important shift occurred in the twelfth century, consisting in moving away from a concept of ‘hermeneutical Jews’, and that this was largely due to the fact that Christendom had to face Islam. As a result, authors increasingly entangled Muslims and Jews in their texts and imagination.^52^ Martin’s work, as we will see, is an important exponent of this development. And yet, we encounter difficulties when it comes to interpreting specific sermons, since these protean texts were designed for being easily adaptable for different occasions and audiences.^53^ Since a sermon manuscript was available to preachers at a particular time and place, its texts were likely used multiple times, and it would be highly surprising if this did not comprise occasions concerned with crusading. Therefore, whenever the crusade appears as a potential reading of a text, I argue that it is justified to read it in this way. Even if an element such as Jerusalem had a metaphorical or spiritual meaning, it is likely that precisely this imagery was chosen because of the historical context in which the text was placed.

First, the *Liber’s* prologue announces the goal of contesting the perfidy of the Jews (*Judaeorum perfidia redarguitur*), mirroring a crucial concern that the Christians had with the Jews for their rejection of Christ (PL 208: 31).^54^ This is a position that runs through the *Liber*: a search for judae* yields 1554 occurrences, but with uneven distribution. The anti-Jewish agenda intensifies considerably in a part of the exceptional *Sermo 4* that can be labelled as an anti-Jewish treatise (the sections 2–28; PL 208: 106–405). This anti-Jewish agenda represents the nucleus around which other subjects assemble, including the other target groups. It tackles essential matters including the elect status of Christians, believed to have been inherited from the Jews; the nature of the Holy Land, which therefore belongs to the Christians; and the typological relationship of the Old and New Testaments, where the latter is understood as a fulfilment of the former.^55^ The Jews in the *Liber* may occasionally come close to being a hermeneutical construct, yet many references to them are certainly to actual groups – though interestingly, not necessarily Jews. *Sermo 22* (Maundy Thursday) calls its doubtlessly Christian audience repeatedly ‘Jews’ (*iudaei*), obviously with a pejorative impetus. As has been discussed elsewhere, this suggests a potential loss of the Christian elect status, following the Jewish precedent; and this represents Martin’s explanation for Jerusalem’s loss in 1187. Even though other preachers shared this radical idea, his rhetorical form is unique. This text thus fuses the Jews into a sermon concerned with calling its audience to participate in the Third Crusade.^56^

The *Vita* corroborates Martin’s anti-Jewish agenda: it narrates how he admonished his fellow clerics to carefully guard the distinctions between Christians and Jews, identifying the latter as *filii diaboli*, the sons of the devil (PL 208: 18). Similarly, it relates how the kings of Aragon and Castile destroyed what is identified as the Jewish castle (*castrum Judaeorum*) at León’s outskirts in 1196. Martin is reported to have assured the inhabitants of León that this would not happen to them, since God protects the Christian city (unlike the Jewish castle).^57^ The *Liber’s* interest in the Jews is thus corroborated by historical action on which the *Vita* reports, action which Martin may have guided or accompanied with his preaching. His consolation of the inhabitants includes a quotation of a sermon-like speech, where he even appears as God’s prophet.^58^ The appearance of Jews in the *Liber*, however, cannot be connected to a specific historical action, since anti-Judaism was widespread throughout the West. Nevertheless, this outstandingly anti-Jewish work, together with other contemporary sources from the kingdom of León, encourage the scholarly trend of questioning the traditional notion of *convivencia*.^59^ The local context shows, therefore, that speaking of ‘hermeneutical Jews’ alone does not fully explain Martin’s work. His Jews are both real and hermeneutical; the two dimensions often go hand in hand or are even indistinguishable.

Second, the prologue announces the goal of exposing and destroying the depravity of the heretics (*haereticorum pravitas detegitur atque destruitur*) (PL 208: 32). This concern surfaces on numerous occasions: haereti* appears 508 times in the *Liber*, and notably in another part of *Sermo 4* that can be described as an anti-heretical treatise (sections 29–38; PL 208: 405–549). This work draws inspiration from Isidore of Seville’s treatment of several heretical groups, but whereas Isidore gave no more than two lines to each group, Martin devoted an entire section to each.^60^ This substantial treatise betrays his expertise in the field, though the work is not introduced by any paratext in the edition or in the manuscript  – likely the reason that modern scholarship has not noticed it. Yet the manuscript does have paratextual framings introducing the different sections, each as a gloss in red ink, for example, *hoc loco convincuntur manichei heretici* (MS 11/1, fol. 150r). The exceptional *Sermo quartus in natale domini* thus hides under this enigmatic heading both an anti-Jewish and an anti-heretical treatise, with its section 29 fulfilling the purpose of a transition by dealing with both groups.^61^ However, the reason why these works were united under the heading of a sermon remains unclear. Perhaps another label would have hampered an otherwise consistent paratext; perhaps both treatises can be considered as repositories of preaching material nonetheless.

The anti-heretical treatise covers, for example, the *Arabici haeretici*, the Arab philosophers (section 37; PL 208: 537–43), who are introduced by the gloss: *hac disputatione convincuntur luciferiani et arabici heretici* (fol. 206v). This label intertwines three of the four target groups – Muslims, heretics and philosophers – a fact that Martin addresses in a passage that also includes the Jews (PL 208: 540). Such entanglements are common throughout the *Liber* and stand firmly in the Latin tradition of the time: the various groups thus become part of the forces of evil.^62^ Martin blames the Arabs for proposing that the soul has a carnal nature and he calls for their total annihilation (*quid aliud agit nisi quod vestram mendacissimam sectam ex toto destruit?*) (PL 208: 541). This is certainly not a hermeneutical discussion. The treatise also has a section on the Cathars (*cathari*), the label for the heretics prevalent in southern France (no.33, part II; PL 208: 494–508; and for the introduction gloss, fol. 184v). He accuses them of rejecting the practice of penance, outlining elaborately why penance is important, and then calls once again for their destruction (*vestra mendacissima secta ex toto evacuatur*) (PL 208: 500). A second issue broached is that the Cathars prohibit widows from marrying again. Martin uses this opportunity to underline the general merits of matrimony (PL 208: 500–08; see also below).

The *Vita* recounts that Martin encountered heretics in southern France when returning from the Holy Land: it presents this episode as a devilish temptation that he repels successfully (PL 208: 14). An interest within the *Liber* and an activity recorded in the *Vita* once more corroborate one another. The anti-heretical material may even have been penned or used on the occasion narrated in the *Vita*. This effort mirrors that of his reform colleagues such as Alan of Lille, active in southern France in the 1190s. Alan’s *Contra Haereticos* applies almost the same division as Martin’s prologue: it has four books devoted to Cathars, Waldensians, Jews, and *pagani* (that is, Muslims) respectively. And in agreement with Martin, it offers a chapter dedicated to the Cathar rejection of penance (PL 210: 352–56).^63^ As Lucas of Tuy’s own anti-heretical treatise demonstrates (c.1235), the label of heresy was also easily transferred to the Iberian context.^64^ Pope Celestine III had, after all, initiated an anti-heretical crusade against Alfonso IX of León (1196), since the latter had agreed on an alliance with the Almohads – *pagani* in the papal document – against the other Christian princes.^65^ As discussed, Martin was close to the king and his queen. The close relationship eventually prompted Pope Innocent III to excommunicate Martin, and he died in this state – of course, not a word about this appears in the *Vita*.^66^ Martin likely disagreed with both the crusade against Alfonso and his king’s Muslim alliance. These delicate circumstances explain why the *Vita* conceals his extensive anti-pagan ambitions (see below), which would have been difficult to sell by reason of his loyalty to Alfonso. The only occasion where the events of 1196 surface is the already mentioned incident when the kings of Aragon and Castile destroy the Jewish castle (an outcome of this crusade), though not a word is said about the Muslim alliance. It is worth returning to the fact that the *Liber’s* manuscript was composed after 1197. In light of the events addressed, it may have been a joint attempt by Martin and his king to re-establish León’s reputation by showing how much the kingdom was devoted to anti-heretical and anti-Muslim action.^67^ This may have been fuelled by the king’s marriage in 1197 to Berengaria, the financier of the *Liber*, whose father, Alfonso VIII of Castile, was a devout crusader.^68^ Significantly, the *Liber’s* ambitions around 1200 foreshadow Alfonso IX’s later career (after 1212), which witnessed major conquests of Muslim territories in the south.

Third, the prologue announces the goal of spurning the teachings of the philosophers (*philosophorum dogma contemnitur*) (PL 208: 32). Searching the *Liber* for philosoph* yields only 45 results, but these include meaningful discussions, notably in a section of the anti-heretical treatise devoted to the ‘heresy’ of the ancient philosopher Heraclitus (no.33, part I; PL 208: 482–94). This polemic attacks a contemporary group by using repeatedly the vocative (*o Heraclitae haeretici*), that is, ‘you heretics who study the works of Heraclitus’. This is also apparent in the gloss that introduces it: *hoc loco convincuntur heraclitis hereticis* (fol. 178r). Martin faults this group for rejecting the institution of matrimony, while describing the bond between Christ and Church as conjugal. And he calls twice for utterly destroying this heresy (inter alia: *ad catholicam etiam fidem magis magisque roborandam, et vestram mendacissimam sectam omnino destruendam*) (PL 208: 492). All this shows that he took issue with another branch of the early university – the scholastics and their interest in philosophical works and unorthodox methods – and it perfectly agrees with the polemics of other reformers.^69^ The philosophers are thus included in the pandemonium of evil, being put on a par with other heretics, Jews and Muslims. For example, it was not a coincidence that Bernard of Clairvaux (c.1090–1153) attempted to condemn Gilbert of Poitiers (c.1080-1154) as a heretic precisely when the forces of the Second Crusade were struggling in the East (March 1148). As Bernard makes clear, this was part of a holistic effort; the goal was to acquire God’s favour on various frontiers so that He would support the crusaders in the Holy Land.^70^ The question remains of where and when Martin, for his part, would have promulgated such action. Unlike for the other groups, we cannot grasp anything specific, but we can take it as evidence for his time in northern France.

Fourth, the prologue announces the goal of annihilating the superstition of the pagans, that is, the worship of idols (*paganorum superstitio, id est idolorum servitus, deletur*) (PL 208: 31–32). This subject appears on numerous occasions throughout the *Liber*, via a variety of terms: pagan*, 43 times; natio*, 53; ismael*, 22; idol*, 165; gentil*, 204; and especially gent*, 957 (thus including *gentiles* and the more ambiguous *gentes*). There are, then, at least some 480 references to pagans, but considering that *gentes* is often also used with that meaning, the number is certainly higher.^71^ Once again, it is evident that Martin provides material for agitating against an actual group, with the Muslims those most commonly labelled as ‘pagans’ at the time, as in the papal letter discussed above. As John Tolan has asserted, the belief that they were idol worshippers persisted until the seventeenth century, despite the available sources that could have told authors otherwise.^72^ Peter the Venerable (c.1090–1156) had already written more accurate works on Islam; these, however, were barely received beyond Cluny.^73^ In his last years, Martin was not far away from Muslim dominions, but perhaps distant enough to uphold such a biblically coloured image of this group. The same has been demonstrated for art historical sources and historiographical writings from León.^74^ Richard Southern rightly asserted an ‘age of ignorance’, underlining that the period subsequent to the First Crusade did not bring an increase in knowledge about Islam. Katherine Allen Smith has suggested that this was also a conscious strategy to label warfare between Christians and Muslims according to the Old Testament model of Israelites versus pagans.^75^ But even if Martin had more substantial knowledge, it is meaningful that he decided to conceal it in his texts. His perception of a pagan enemy complies perfectly with a tradition of depicting Islam that originated north of the Pyrenees, since Iberian texts often took a more nuanced approach, such as by designating different Muslim groups via different ethnonyms, while using the various terms for ‘pagans’ comparatively rarely.^76^

Martin’s crusading interests also surface in frequent discussions of Jerusalem and the Holy Land – even though these elements require a consideration of the four senses of Scripture and do not necessarily relate to crusading.^77^ Yet, at least three of his sermons can be placed in the specific context after 1187 and are thus highly pertinent to crusading: *Sermo 18* (Palm Sunday); *Sermo 22* (Maundy Thursday); and his *De sancta cruce* (*De sanctis, Sermo 5*). The first two broach Jerusalem’s fall in 1187; and the last one belongs to a genre that emerged in reaction to the loss of the True Cross in the same year. These three sermons testify to Martin’s activity in the Third Crusade, an activity indicated in the *Vita* by his journey to the Holy Land and his service there for the Hospitallers (PL 208: 13).^78^ He also penned a strongly historical commentary on John’s Revelation (a part of the *Liber*), discussing, inter alia, the journey of the Last World Emperor to Jerusalem. This probably dates to the time of the Third Crusade, given that he places this discussion in the comment on Rev. 11, a chapter that deals with a pagan occupation of Jerusalem. Many other preachers drew on this reference for endowing the city’s loss in 1187 with providential meaning.^79^ The apocalyptic nature of crusading is also apparent in the regular occurrence of pagans and heretics in the same commentary. The *Liber* contains numerous further elements that are potentially crusade-related: ideas of militant martyrdom, the promise of *plena indulgentia* (likely a reference to the papal crusade indulgence), and the frequent use of militant imagery.^80^ The ceaseless occurrence of such motifs suggests that Martin’s preaching had the goal of initiating or accompanying military action, which is concordant with the analysis above that the *Liber* was devoted to agitating against four actual groups. ‘Pagans’ as a label for the Muslims, however, can indicate both the Iberian Peninsula and the Holy Land as a theatre of operations, an issue that will be addressed in the next section.

## The Third Crusade, Popular Preaching, and the Liturgical Front: Three Case Studies

A close analysis of three texts should substantiate Martin’s involvement in the crusade effort and elucidate the nature of his ideas. The first text can indeed be considered as a sermon, specifically devoted to initiating or promoting crusade efforts. The other two are among the lengthier and hence more enigmatic ‘sermons’ of the *Liber*. According to their paratexts, they were still meant as repositories of preaching materials, yet, in their form and length, were not suitable for being directly preached. Similarly, the crusade purpose surfaces in these two in a more protean fashion: while we will encounter passages clearly concerned with warfare against the Church’s various enemies, other passages could equally refer to spiritual warfare and the other senses of Scripture. Identifying ‘the crusade’ and the literal sense of Scripture will thus be an essential concern of the following pages.^81^

### Sermo 18: In ramis palmarum

*Sermo* 18, the first of the three texts here under discussion, is ascribed to Palm Sunday. Jessalynn Bird has argued that this feast strongly lent itself to the purpose of crusade preaching, since it concerns Christ’s entry into Jerusalem and thus offers a role-model for pilgrims and crusaders.^82^ Appearing in the second part of the first codex (that is, after the start of the new foliation), it initiates a sequence of three Palm Sunday sermons. It is the longest of the three, though still of a size suitable for oral delivery, and it also has the strongest occurrence of crusade-related motifs. As the appendix shows, it follows an exemplary pattern, whereby Martin provides two or more texts on the same feast, displaying a high density of crusade elements in one but not in the other(s). As is typical for sermon material, he supplies texts for different occasions and audiences. It is noteworthy that, even though Jerusalem and the Holy Land loom large in *Sermo 18*, pagans do not. This raises two possibilities: either the text remains within an exegetical register (perhaps addressing a monastic, rather than literal, Jerusalem), or it complies with a pattern generally observable in crusade-related sermons. Such rarely take note of the enemy, but focus instead on gaining salvation and on the holy places.^83^

The sermon starts by asserting that Isaiah’s prophecy has been fulfilled in Christ; it also predicted His Second Coming. Anticipating the latter, Martin insists that the Church must be protected against both visible and invisible enemies (*Deus Ecclesiam suam doctrinis et exemplis regendam, et ab inimicis visibilibus et invisibilibus custodiendam commisit*) (PL 208: 811). It is therefore clear from the beginning that this sermon transcends a spiritual register. A call for action substantiates that it does not pertain to a contemplative monastic sphere ([ecclesia] *studens sanctis actionibus, exspectet Jesum Christum Salvatorem et sponsum suum venientem ad judicium*) (PL 208: 811). Martin asserts that the Old Testament city of Bosra (Is. 63: 1) signifies the earthly Jerusalem: the latter has been fortified in imitation of the first (*Hierusalem terrenam significat, quae firmissimis muris fuit munita, in qua passus est Dominus*) (PL 208: 813). He underlines here the location where Christ overcame death. Thus, it is evident that he deals with the actual city – and hence, the crusade (cols. 811–13 and MS 11/2, fols. 99v–100v).

Christ already showed the way for defeating both invisible and visible enemies, the sermon reveals. This is exemplified via multiple references to the Roman conquest of Jerusalem (70 CE), by which Christ punished the Jews for the crucifixion (inter alia: [Christus] *quamvis occisus, calcavi eos, Judaeos videlicet et daemones*) (PL 208: 814). Some lines later, Martin clarifies that Jerusalem’s penalty, induced by God’s vengeful rod, is an act of mercy (*hac enim virga percutit, ut misereatur*) (PL 208: 816). Referring to Hebr. 12: 6 (*castigat Deus omnem filium, quem recipit*), it is the correction of a sinful state, which pertains to the limbs of the Corpus Christi, that is, the entire Church (*membra ejus virga indignationis ad correctionem percutiuntur*) (PL 208: 817).^84^ This makes apparent that the event of 70 CE only provides an example for addressing current concerns. Martin thus develops a theological model for reading contemporary conquests of Jerusalem. Other preachers specifically understood Jerusalem’s fall in 1187 as an opportunity for salvation that God had created, also using Hebr. 12: 6 for this purpose (cols. 814–17 and fols. 101r–102r).^85^

Having stressed once again that all Christians are united in the Corpus Christi (*hoc quoque ad membra et caput aeque refertur*), Martin outlines how God permits that both Jews and pagans are currently afflicting the Church ([deus] *ecclesiam vero tam de Judaeis quam de gentibus multis disciplinarum angoribus affligit*) (PL 208: 818). This is epitomised by quoting 2 Tim. 3: 12 (*quicunque enim volunt pie vivere in Christo Jesu, persecutiones patiuntur*). This is a significant appearance of pagans in the text, betraying Martin’s concern with an actual group, likely the Muslims. The current events have brought a pollution of the Corpus Christi (*sed membra ejus vitiis inveterata corrumpuntur*). Thus, we read repeatedly that this ignominious state must be met with *Imitatio Christi* and martyrdom (inter alia: *pelles* [Christi] *sunt omnes sancti, qui se cum Christo mortificaverunt, quibus teguntur vitalia corporis membra, ut possint imitari Christi passionem*) (PL 208: 818). Calling a sermon’s audience collectively to martyrdom suggests the context of crusading, an arena in which everybody had an opportunity usually only granted to a few elect (col. 818 and fol. 102v).^86^

Martin continues by outlining a menacing sinful state, from which the Church now suffers daily. There is a current situation in need of being addressed:

The Church, which is made from Christ’s flesh and bones, endures these sufferings on a daily basis, so as to add those limbs [to the Corpus Christi] that were absent from Christ’s passion [Col. 1: 24]. Thus, it is said: He has besieged and enveloped me with poison and tribulation [Lam. 3: 5], because the Church is now besieged by all the evils. It dwells among scorpions where Satan’s throne is [Rev. 2: 13]. It is surrounded by an army of heretics. It is drunk from the false brothers who are like poison and tribulation. And the torments of the persecutors oppress it everywhere.^87^

The Church is besieged all around, explicitly by an army of heretics and ‘the persecutors’, likely a reference to the Muslims. This must be brought to an end, hence the appeal to Martin’s audience. Thereafter, he explains several moral implications behind these physical phenomena. This is an essential strategy throughout the text: first broaching a physical matter, that is, the literal sense of Scripture (introduced with *historice*), and then imbuing it with a moral dimension (introduced with *moraliter*). We see thus that he creates meaningful causalities between the senses; they are the opposite of antithetic (cols. 819–22 and fols. 103r–104r).

Underlining once more that the enemies are combatting them both internally and externally, he turns to Jerusalem’s loss in 1187, a powerful manifestation of the external enemies:

It follows: He is a bear lying in wait for me, a lion in hiding [Lam. 3: 10]. Literally: What can be more dreadful than seeing how the creator of our redemption turns into a bear and a lion because of us? The bear is a savage beast, sly in his limbs and most vigorous in his loins. One calls the Lord a bear and a lion with regard to Nebuchadnezzar as well as Titus and Vespasian, who are elsewhere called a boar and unique [Ps. 80: 14]. Through such helpers, the power of God takes vengeance on the enemies. We lament thus rightly that the pious and merciful Lord and God has turned into a bear and a lion because of our sins, in order that his vigour may besiege and afflict his enemies. Jerusalem has been placed as if it is a target for an arrow [Lam. 3: 12], because it is besieged all around by enemies. Thus, as if wounded by arrows, it is frequently forced to return to its saviour. God sent now these arrows, that is, these gentiles, against Jerusalem, like a bow moderately stretched for causing only temperate harm. He thus hoped that you would finally learn to fear him, lest you would die wounded by the arrows of his enemies.^88^

Referring to the Babylonian and the Roman conquests as past manifestations of divine punishment, Martin asserts that the holy city has now been lost *peccatis exigentibus*, the common explanation for the event of 1187.^89^ God sent the pagans as ‘arrows’ against it (*has sagittas, id est has gentes* […] *contra Hierusalem misit*). Reading these lines, it is clear that this is not about the past conquests. A current situation is introduced right at the start: God turns into a bear and a lion ‘because of us’ (*ursum et leonem fieri nobis*).^90^ This sermon was a reaction to Jerusalem’s loss in 1187 and likely meant to entice potential crusaders, as suggested by the idea that God permitted the event as a necessary act of correction and thus an opportunity for salvation. Yet, a few lines later, Martin proceeds to a broader objective, sketching a *corpus diaboli* to which heretics and hypocritical Christians belong.^91^ Explaining the eastern events via *peccatis exigentibus* generated a growing interest in domestic enemies. Martin’s sermon plausibly deployed Jerusalem’s loss as an incentive for combatting heretics, possibly in southern France or León. Considering his biography, both are potential arenas for delivering such a sermon text – and perhaps he did so in both (cols. 822–23 and fol. 104r–v).

Finally, Martin describes Jerusalem’s current conquest as an act of adultery, since Christ, its groom, has abandoned it due to the Christians’ sins – once more, it is clear that this cannot refer to its past conquests.^92^ This is substantiated by other preachers’ sermons using the same meaningful imagery for explaining the event of 1187; and it powerfully connects to Martin’s discussions of marriage in the anti-heretical treatise.^93^ Mentioning once again the city’s earlier conquests, he asserts that the unbelievers will follow the Antichrist instead of Christ (*pro Christo Antichristum sunt recepturi*) (PL 208: 824). This engenders a meaningful interplay: the past events deliver resources for endowing the current conquest with meaning, while the reference to future events shapes expectations in the audience. This imbues the act of crusading with an eschatological outlook, another meaningful parallel: preachers conceived of the Third Crusade in deeply apocalyptic terms.^94^ It mirrors the argument from the sermon’s beginning, which was focused on the Second Coming (cols. 823–24 and fol. 105r).

At the end, Martin quickly recapitulates several subjects, apparently as a reminder of what the important points of his sermon were. These include the idea of a still incomplete Passion (*his et hujusmodi doloribus totum Christi corpus usque ad consummationem cruciabitur*), idol worship, heretics and the *corpus diaboli*, since Satan operates with the help of his ‘satellites’ (*sic et Redemptor noster contra mundi principem et satellites ejus dimicans et superans*) (PL 208: 825). He also includes a call for *Imitatio Christi* that harnesses 1 Pet. 2: 21, even inviting his listeners to die like Christ, a strong indicator for a crusading context (*ejusque vestigia, in quantum possibile est sequamur, et cum eo, despectis hujus mundi blandimentis, moriamur*) (PL 208: 826). This reference, found among many crusade preachers, evokes an *Imitatio Christi* specifically in the Holy Land (cols. 824–26 and fols. 105v–106r).^95^

In conclusion, it is clear that this sermon is concerned with crusading. The text tackles the conquest of 1187 twice; and the literal sense of Scripture surfaces repeatedly by referring to both the earthly Jerusalem and the various enemy groups: pagans, heretics, false Christians and Jews. This ensemble perfectly mirrors the *Liber’s* prologue. The multiple references to domestic enemies suggest that the sermon takes the event of 1187 as a starting point, but indebted to the logic of *peccatis exigentibus*, it was more devoted to initiating action on the anti-heretical frontier, likely after the Third Crusade had failed. This complies with the activities of other preachers such as Alan of Lille, who penned a similar sermon: therein, the Holy Land’s current condition likewise represents an incentive for combatting heretics.^96^ Martin also shows numerous parallels with his confederates, including biblical references such as Hebr. 12: 6 or 1 Pet. 2: 21. These results substantiate that he belonged to the Parisian network.

### Sermo 11: De actibus apostolorum

The subject of the second case study bears the title of *Sermo de actibus apostolorum* (in the edition: *De diversis, Sermo 11*), but it is simply too long for a sermon (MS 11/3, fols. 21r–38r).^97^ Regarding its structure and rhetoric, it does not read like a model for oral delivery either. It is a treatise-like text that circles around the theme of popular preaching, as epitomised in the example of the Apostles. It is the fourth text in the second codex, preceded by three sermons on Easter and succeeded by one on the second Sunday after Easter. The latter focuses on Jerusalem, though lacking specific references to historical dimensions. *Sermo 11* outlines the necessity for and purposes of popular preaching in a strongly explanatory manner, for an audience still unfamiliar with the Parisian agenda. Martin is here instructing the canons of San Isidoro. He elaborates on many of the points tackled in the *Liber’s* prologue: *Sermo 11* demands repeatedly that the canons teach others *verbo et exemplo*, underlining that what they preach must concur with their own lifestyle (PL 209: 137–38, 149, and 181). One section is specifically concerned with the necessity of preaching to simple people in a plain and clear manner (*curaverunt summopere rudibus populis plana et capacibilia, non summa et ardua praedicare*). Preachers should thus consider their audience’s capacities (*praedicatores igitur sanctae Ecclesiae juxta audientium capacitatem, ut dictum est, debent praedicare*) (PL 209: 164–66). This offers evidence that Martin was aiming at popular audiences beyond the secular elite. Moreover, the text more than once entangles preaching and warfare, just as it interlocks *docere* and *roborare*, that is, strengthening or defending the Christian faith, another concept familiar from the prologue (PL 209: 139, 143–45, 152, and 179–80). All these are prescriptions for leading the opposite of a contemplative life. Indeed, Martin calls explicitly for a *vita activa*: preaching stands here in the service of initiating and accompanying military action (PL 209: 146 and 183). *Sermo 11* evidences his attempt to implement the Parisian programme in the diocese of León, where, as discussed, he could draw on an extensive parish network.

The concept of *Vita Apostolica* had been transferred to the crusading arena since the days of the First Crusade; sources even identified the crusaders as ‘the sons of the Apostles’.^98^ In agreement, *Sermo 11* is rich in militant imagery and references to pagans and Jews, Jerusalem and martyrdom. The crusade surfaces, for example, in a passage that formulates an eschatological vision of violence: ‘There will only be one name of the Lord [Zach. 14: 9], after the vicious religion of idol worship has been crushed. Then, the whole land will be restored [Zach. 14: 10], the land bordering onto the desert, in which the Jews had once lived.’^99^ Annihilating paganism is presented here as a precondition for regaining the Holy Land, that is, fulfilling the prophecy of Zach. 14, a meaningful incentive to action. This draws on the common nexus between combatting Islam and eschatological beliefs.^100^ It is a powerful example of how Martin uses biblical elements for reading current phenomena. He likely tackles here the situation after 1187, when large parts of the Holy Land had been lost to ‘the pagans’. Another passage mirrors the prologue in assembling the enemy groups: heretics, false Christians, the ‘pagan persecutors’ (*persecutores pagani*) and the Jews.^101^ In agreement with the Palm Sunday sermon, he constructs a *corpus diaboli*, noting a few lines later that they can all expect their damnation at the imminent Last Judgement.

After having outlined the preaching programme at the beginning of *Sermo 11*, Martin develops an elaborate militant vision. First, he presents the Old Testament Gideon as an example for the *milites Christi* and their martyrdom (*hos invincibiles Christi milites, sanctos scilicet apostolos et martyres, Gedeon fortissimus virorum praefiguravit* [Jud. 7]). God appears as their warlord, guiding them in battle (*Dominus, scilicet, fortis et potens: Dominus potens in praelio* [Ps. 24: 8]). Gideon was therefore victorious despite leading a force of only 300 men (PL 209: 143). Second, Martin connects the Tau from Ez. 9 with the cross, highlighting that this pertains to ‘the cross itself’ (*non jam crucis species, sed ipsa crux esset*), which grants victory over the enemies (*ferrum hostium crucis ligno superetur*). These lines strongly indicate the specific relic of Jerusalem; Ez. 9 was a key reference for endowing its loss in 1187 with meaning.^102^ Alluding to Mt. 16: 24, Martin even encourages his audience ‘to take the cross’, a strong indicator for crusading (*qui sequentes Christum tanto verius crucem tollunt*) (PL 209: 144).^103^ Third, inspired by Rev. 19, he delineates that Christ leads his soldiers into battle against the enemies of the Christian faith (*hi, Christo duce, contra hostes fidei ad praelium pergunt*). The designation as *hostes fidei* manifests clearly that this is not about spiritual warfare; several hints have now surfaced that *Sermo 11* lends itself to physical action. It is specifically concerned with the role of *preachers*, consequently asserting that Christ’s soldiers are accompanied by the trumpets, that is, the roar of preachers (*designatur itaque in tubis clamor praedicationum*). It also repeatedly entangles preaching and martyrdom (inter alia: *praedicaverunt martyres, donec eorum corpora in morte solverentur*) (PL 209: 144–45). Martyrdom is presented here as a preacher’s goal, a remarkable idea that likely indicates the redemption gained via crusading.^104^ This seems logical because a crusade army needed preachers, who should thus be motivated to participate. It has become clear that annihilating paganism, following Christ into battle, and becoming a martyr – in other words crusading – are essential cornerstones of Martin’s programme of popular preaching. These are ideas very much present in sermons of other preachers; here, we see Martin trying to implement them in a new context by instructing his still inept brothers.

### Sermo 29: In rogationibus

The subject of our third case study, *Sermo 29*, was not a model for oral delivery either (MS 11/3, fols. 106v–126r). As its first lines make clear, it is a treatise *De officiis,* specifically concerned with rogation liturgy (PL 208: 1035). It appears in the second codex, preceded by another rogation treatise, which discusses basic purposes of such a liturgy, while not focusing much on crusade-related themes. This is a familiar pattern: Martin provided two texts on the same subject, one crusade-related, the other having another purpose. Yet, the two share the objective of instructing uneducated clerics, especially by frequently underlining the necessity of preaching, very much in agreement with *Sermo 11*. Importantly, *Sermo 29* elucidates how liturgical actions were capable of connecting the diverse crusade arenas in which Martin was involved. Referring to *peccatis exigentibus* for an explanation more than once in the *Liber*,^105^ Martin calls on Christendom not only to wage war in the East, but also to deal with the issues within, and by such action to secure God’s support on the Holy Land frontier. Thus, he urges Christians to combat heretics and other inner enemies (as in the Palm Sunday sermon), as well as to diminish their own sins via penance and liturgical practices. The recurrence of failure in the East, critically after 1187, made both warfare against inner enemies and crusading’s liturgical support into frequent features of western religious practice.^106^ One could pursue such liturgical support from the distance, as for example, by a person in the West praying for the crusade’s success in the East.^107^ Exactly this logic is exemplified in Martin’s *Sermo 29*: it discusses how and why his audience should support the crusade via such actions (though the vast treatise does not remain limited to this). In agreement with the subjects of my other two case studies, this text also stresses repeatedly the nexus between spiritual and physical enemies, leaving no space for a purely hermeneutical interpretation (PL 208: 1059, 1063, and 1083–84).^108^ As a result, crusade efforts in Iberia may have had the objective of initiating remote liturgical support. This was one measure for averting God’s whips and an attractive option for those who did not participate personally in a crusade, including Martin’s brothers in San Isidoro, the *Liber’s* addressees.

The beginning of *Sermo 29* stresses that rogation liturgy has the essential purpose of gaining God’s support against ‘the enemies of the Christian religion’ (*ut ergo Deus haec omnia avertat, et nos defendat atque eripiat ab intemperie aeris, a bellis et ab hostibus Christianae religionis, facimus has litanias*) (PL 208: 1038). Such a broad mandate thus straddles all enemy groups and several crusade arenas. In the same context, Martin presents the Old Testament city of Nineveh as a model for penance (*jejunium ad similitudinem Ninivitarum*), but also as a warning, since it was eventually destroyed (PL 208: 1037).^109^ Later, he repeats that pagans (*gentes*) have invaded Nineveh (Soph. 2: 13–14), an antetype that raises the necessity of killing all those by the sword who do not do penance. This shall guard ‘the pathway of the tree of life’, a synonym for the cross (*tenebrarum colorem nolunt per poenitentiam abluere, quod interficiendi sunt illo gladio, quo utitur ad custodiendam viam ligni vitae*) (PL 208: 1049). This reminds us of the vision of erasing paganism that we encountered in *Sermo 11*. A few lines later, Martin addresses a current situation: the Christians have been cast out of the Holy Land, sojourning now in the adjacent regions (*corpus itaque nostrum aut in Aegypto, aut in Babylone, aut in Syria, aut in Palaestina, aut ubicunque est*). The Corpus Christi is dispersed in exile, instead of being united in Jerusalem. Referring to Ps. 137: 4, this makes it impossible to celebrate the liturgy (*quomodo cantabimus canticum Domini in terra aliena?*). These lines strongly suggest the situation after the events of 1187 (PL 208: 1050). Martin underlines thereafter that the liturgy can only be truly celebrated in the holy city (PL 208: 1051), likely denoting the liturgy of Latin Jerusalem that had commemorated the conquest of 1099 – until 1187.^110^ His audience is now called to replace it via frequent rogation services.

Another part of the treatise takes the Holy Land’s current condition as an incentive for broaching wider issues – a strategy familiar from the Palm Sunday sermon. Martin heralds these passages by asserting that preaching to the people must show ‘the path to salvation’ (*viam salutis populo Dei ostendere debuimus*), once again making clear that his efforts targeted broad popular audiences. Yet this is currently hampered by the fact that the gates to Zion have been destroyed (*portae jacent destructae* [Lam. 1: 4]), just as Jeremiah’s Lamentations have predicted (*Jeremias in spiritu praevidens*). This is a meaningful image used by several other authors for describing the events of 1187.^111^ Following the common explanation, Martin rationalises that God sent the enemies as punishment for sins (*adversa, quae pro peccatis nostris justo Dei judicio patimur*). The Corpus Christi has been polluted (*omnia membra, subjecti scilicet populi, inficiuntur, id est patiuntur*); and the preachers failed hitherto to instruct the simple people (*Ecclesiasticorum quippe virorum culpa simplex populus Dei plerumque patitur flagella*) (PL 208: 1062–63). Then, he concludes:

When the shepherds of the people do not clearly outline the righteous path, those who follow them will go severely astray. […] As a result, the Church is bitterly aggrieved because of the sins of both its shepherds and its subordinates. It happens thus frequently that famine befalls it, that the pagan people invade its borders, that the ferocity of the wolves dares to mutilate its human limbs, and that the depravity of the heretics seeks to seduce its children and to infect them with the poison of their error.^112^

The failure of the preachers is the reason that the Church *peccatis exigentibus* is now attacked by the pagans on several frontiers (*pagana gens illius terminos invadit*), just as it suffers other severities, including heretics.^113^ This reflects Martin’s personal background, which knew both pagan frontiers, the Holy Land and Iberia. His argument evokes a holistic war against paganism, likely inspired by what Bernard of Clairvaux had propagated on the Second Crusade’s eve. Pursuing ambitions simultaneously in the Holy Land, eastern Germany, and Iberia, Bernard believed that only such an all-encompassing effort would secure God’s support in the East.^114^ That Martin shared this idea is apparent in his multiple statements calling for a total annihilation of paganism, starting with the *Liber’s* prologue.^115^ This prospect was also promulgated in two letters that Pope Clement III addressed to the archbishop of Toledo and his suffragans on the Third Crusade’s eve (May and June 1188). Referring to the original crusade encyclical *Audita Tremendi* (October 1187), these granted crusaders in Iberia the same remission of sin (*eandem remissionem*) as those participating in the eastern expedition.^116^ The belief was that a second frontier would actually affect the one in the Holy Land, since God was the driving force behind all these events – and one could try to please him via rogation services.

There is consequently a potential for interweaving Holy Land and Iberia, practically and ideologically.^117^ However, it is remarkable that in no instance of which I am aware does Martin explicitly distinguish between the two frontiers, despite his concern with pagans and crusading on so many occasions.^118^ In agreement with the papal letters, he apparently understood the two theatres to be part of the same cosmic struggle. The *Liber* holds three types of relevant occurrences: (1) a conjunction of Holy Land and pagans, which evidently indicates the eastern crusade; (2) the Holy Land without pagans (as discussed below); and (3) pagans without Holy Land, a finding that raises the possibility that such cases are concerned with Iberia. Yet details that would relate to the Peninsula remain rare throughout: either Martin did not have the opportunity to add such, or he did not deem this necessary. This encourages the hypothesis that the *Liber’s* texts originated largely beyond the Peninsula’s borders, though once back in León, he possibly recycled them for agitating against local Muslims. Contesting previous scholarly ideas, Nikolas Jaspert has shown that the Iberians cared very much about crusading to the Holy Land. Other scholars have unearthed the providential nature of warfare within the Peninsula.^119^ This arena thus offered many opportunities for crusade-like action and preaching, even if they were still hampered by the conflicts between Iberia’s Christian princes.^120^ Martin’s ideas shed light on the providential entanglement of Iberia and Holy Land; these informed the organisation and direction of crusade efforts. Two indications bear witness to his intention of adopting the *Liber* for the home front: the manuscript, whose production was facilitated by specific interests in León; and the prologue, which calls the canons of San Isidoro to fulfil the formulated goals. Martin also added a few sermons in his last years, including two that hint at Alfonso IX’s incestuous marriage with Berengaria, a critical issue of the late 1190s.^121^ Similarly, in 24 cases, Isidore appears as *patronus noster* (the patron of San Isidoro); one passage relates him to the town of León (*beatum ergo Isidorum Legionensis urbis specialem patronum*) (PL 208: 408). All this indicates that Martin intended to repurpose the *Liber* for the local context.

It seems symptomatic that the *Liber* cares so much about the Muslims, where other crusade preachers did not pay them much attention at all in their sermon texts.^122^ This likely reflects the adaptation for Iberia, where not so much the reappropriation of holy places, but the defeat or expulsion of Muslims was at stake.^123^ However, it is also a blending of genres, straddling sermon material and polemical treatise. This makes for an idiosyncratic opus, which can be described as an attempt to reassert the religious alterity of Islam (one among other objectives).^124^ Martin may have deemed this an urgent issue after his return to the Peninsula, where he suddenly found Muslims much closer to Christians than in the rest of Europe. This hypothesis is corroborated by considering where exactly ‘pagans’ appear in the *Liber*. They remain largely absent from the sermons that can be related to the Third Crusade; these agree thus in outlook and focus with those of other preachers. Vice versa, passages where Martin discusses pagans more elaborately suggest that he had first-hand acquaintance with them. For example, a section of *Sermo 4* (part of the anti-Jewish treatise) explains their dark skin as a sign of their sinfulness – and yet it remains difficult to determine the Iberian context.^125^ The divergence between contents on the one hand and the work’s final shape on the other offers evidence of how Martin adopted materials which predated his return to León. As *Sermo 11* made clear, he planned to promote popular preaching and clerical reform in the Peninsula, thus foreshadowing what preachers and papal legates would pursue in subsequent decades.^126^ Yet due to political circumstances, notably the difficult relationship between the pope and the Leonese king, Martin’s efforts may have fallen on deaf ears, despite indications that the king himself supported him. What Martin envisioned for Iberia, critically in *Sermo 29*, was a complementary frontier that supported and prepared the eastern endeavour in spiritual and providential terms. It is a plausible hypothesis that the failure of the Third Crusade refocused his attention on the sins within Christendom – this would mirror the shifting efforts of other preachers such as Alan of Lille. As a result, he returned to León, where he repurposed the *Liber* by targeting various groups present on the multi-confessional Peninsula. Agreeing with this logic, then, Innocent III understood the victory at Las Navas de Tolosa as a divine sign: the cleansing had been successful; they were now entitled to prepare a new expedition to the East.^127^

## Conclusions

Three essential ideas shaped the form and purpose of Martin’s work, just as they entwined distant crusade arenas. First, the explanation for failure in the Holy Land redirected efforts towards the corruption within, both of the Christians themselves for their own sins and of the enemies among whom they lived: heretics, Jews and false believers. Martin covers all these groups in his great work and explicitly entangles them on numerous occasions. His piece on Palm Sunday (*Sermo 18*) even took the fall of Jerusalem in 1187 as an incentive for combatting heretics. Second, liturgical actions supported and prepared crusade endeavours: disregarding any spatial distance, they offered attractive options for non-participants. Thus, the work’s final version is addressed to Martin’s brothers in San Isidoro: these potential preachers were responsible for an extensive parish network, in cooperation with the bishop and the king of León, and consequently had the opportunity to implement the *Liber*. These efforts are epitomised in both a vast treatise on rogation liturgy (*Sermo 29*) and an elaborate tract on popular preaching (*Sermo 11*), both of which significantly incorporate the crusade. This evidence undermines any simple identification of crusading with warfare: crusading adhered to a much broader effort, a penitential and liturgical practice essentially entangled with preaching. *Sermo 11* made clear that Martin did indeed aim at a broad popular audience, and not only at León’s elites. The same text proposes more than once a radical idea: that preachers should become martyrs. This certainly reflects a crusading context rather than daily life in a Leonese parish (even though the parish may have been the point of departure for this journey). Third, the idea of a holistic single war against paganism made the Holy Land and the Peninsula into two inseparable frontiers. Thus, Martin does not see any need to distinguish the two throughout his many pages, even though he is frequently concerned with ‘the pagans’. While *Sermo 18* represents a proper sermon, close to orality and ready to be preached, *Sermo 11* and *Sermo 29* are treatises, more diverse in their contents. These aimed at educating preachers, specifically the canons of San Isidoro and perhaps other clerics in León, and provided them with rich materials. In conclusion, the crusade was an important principle in shaping and organising the *Liber*, whose content and form are thus related. The work was an attempt at applying the new methods of organising knowledge developed in the early Parisian university – though perhaps an excessive one at roughly 700 folios.

For modern scholars, Martin’s work shows how worthwhile it is to explore sermon material under the crusade’s lens. It is permeated by crusade-related subjects and crusade-specific ideas – but its paratext is liturgical, which is why it has previously escaped the attention of crusade scholarship. This is most salient in the case of the vast anti-heretical treatise that remained hitherto hidden under the heading of *Sermo quartus in natale domini*. Considering such a work in a crusading context illuminates activities, protagonists and ideas that are absent from the chronicles and have thus remained invisible to scholarship. The liturgical year and the existing parish structures represented the common and most effective way for furthering crusade efforts, whereas assembling large crowds – that is, those events reported in chronicles – was a far more laborious endeavour. This has important implications for crusade scholarship: we must not limit ourselves to chronicles mentioning historical actions, for sources such as sermons offer vivid evidence for the fact that preaching and crusading took place in a particular local context. The stories that sermons deliver can thus complement, and often even modify, those that have been told to date on the basis of the chronicles.

A close analysis of Martin’s work demonstrates how ‘the crusade’ surfaces in such texts, notably via specific enemy groups and the literal sense of Scripture. It has become clear that the construction of artificial boundaries between the senses of Scripture does not help us. The *Liber* underlines on numerous occasions that literal and allegorical were intrinsically entangled, as were physical and spiritual enemies. Even if we cannot pin down the crusade purpose in the specific text, a sermon was likely used multiple times, thus probably including occasions concerned with crusading. Identifying the crusade in Martin’s texts was essentially permitted by unearthing manifold parallels with other preachers, predominantly from the Parisian network. Coming to terms with the fact that medieval Latin did not have a crusade-specific terminology, these results offer new methodological tools for investigating the innumerable sermon texts that still remain untapped in crusade scholarship.

Mapping crusade motifs throughout the *Liber* has permitted us to reach conclusions about the arenas in which Martin was active – results that are always perfectly corroborated by his *Vita*. The appendix, which presents this data systematically, allows other scholars to further explore this rich work. For example, a detailed study of the anti-heretical treatise would certainly be worth doing. As things stand now, it seems clear that Martin was active against (1) philosophers, placing him in Paris; (2) heretics, placing him specifically in southern France; (3) Jews, questioning traditional ideas of *convivencia* in the Peninsula; and (4) pagans, that is, Muslims, entangling him with both the Holy Land and Iberia. Whereas some texts can be related to the Third Crusade and its aftermath, the work’s prologue and the physical manuscript evidence place it in León. This article has also assembled some indications that the *Liber* was a collective project of Martin, the other canons, and the Leonese court. The latter apparently had an interest in presenting itself as a crusader kingdom, probably reacting to the crusade that Celestine III had waged against Alfonso IX because of his Muslim alliance. This would fit in perfectly with dating the *Liber’s* manuscript between 1197 and 1203. The interplay of this historical context and the ideas present in the *Liber* suggest that the work was assembled for purposes on the Iberian home front in Martin’s last years (and plausibly served a purpose beyond his death) in an effort to prepare a new expedition to the East, yet we see how it reflected his earlier activities and whereabouts. Martin may even have prepared the ground for – or at least his work represents an important anticipation of – various crusading and similar phenomena that would fully unfold in the Iberian Peninsula a generation later.

## Footnotes

^1^ For previous considerations, see esp. Adeline Rucquoi, ‘L'invective anti-juive dans l'Espagne chrétienne. Le Sermo IV, In natale Domini II, de Martin de León’, *Atalaya* 5 (1994): 135–51; Raymond McCluskey, ‘The Genesis of the Concordia of Martin of León’, in *God and Man in Medieval Spain*, ed. Derek W. Lomax and David MacKenzie (Aris & Phillips, 1989), 19–36; Amélie de las Heras, ‘El Contra Judaeos de Isidoro de Sevilla en la predicación regular ibérica del final del siglo xii. Entre identidad confesional y estaturia’, in *Christian, Jewish, and Muslim Preaching in the Mediterranean and Europe*, ed. Linda Gale Jones and Adrienne Dupont-Hamy (Brepols, 2019), 147–74; de las Heras, ‘Martin de León († 1203) et la culture scolaire ultra-pyrénéenne. Les Sentences de Pierre Lombard dans la Concordia’, *Memini* 18 (2014): 1–25.

^2^ See Antonio Viñayo González, ‘La abadía de canónigos regulares de San Isidoro de León en el siglo XII: Vida espiritual e intelectual’, in *Pensamiento Hispano Medieval*, ed. José María Soto Rábanos (Consejo Superior de Investigaciones Científicas, 1998), 117–40; Therese Martin, *Queen as King: Politics and Architectural Propaganda in Twelfth-Century Spain* (Brill, 2006); Janna Bianchini, *The Queen's Hand: Power and Authority in the Reign of Berenguela of Castile* (University of Pennsylvania Press, 2012), 49, 52–53.

^3^ See Richard H. Rouse and Mary A. Rouse, *Authentic Witnesses: Approaches to Medieval Texts and Manuscripts* (University of Notre Dame Press, 1991).

^4^ I used primarily the *Corpus Corporum* of the University of Zürich (https://mlat.uzh.ch/home). The counting of word citations comprises all of Martin’s works, and it is always noted how exactly I searched, for example, via *haereti**.

^5^ See Cecilia Gaposchkin, *Invisible Weapons: Liturgy and the Making of Crusade Ideology* (Cornell University Press, 2017), 41–53.

^6^ See, e.g., Christopher J. Tyerman, *The Invention of the Crusades* (Macmillan, 1998), 49–55, 76–83; Christoph T. Maier, ‘When Was the First History of the Crusades Written?’, in *The Crusades: History and Memory*, ed. Kurt Villads Jensen and Torben K. Nielsen (Brepols, 2021), 13–28.

^7^ Alexander Marx, *The Preaching of the Third Crusade (1187–1192): The Early University of Paris, Biblical Exegesis, and the Coming Apocalypse* (Brill, 2024).

^8^ On the complicated nature of narrative sources on preaching, see also Alexander Marx, ‘Multilingualism and Crusade Preaching: The Narrative on Bernard of Clairvaux’s Preaching in Germany’, *Historical Research* (2025, advance version published).

^9^ Jessalynn L. Bird, ‘Heresy, Crusade and Reform in the Circle of Peter the Chanter, c. 1187-c. 1240’ (PhD diss., University of Oxford, 2001); see also John W. Baldwin, *Masters, Princes and Merchants: The Social Views of Peter the Chanter and His Circle*, 2 vols. (Princeton University Press, 1970).

^10^ See Marx, *Early University*; Jean Longère, *Oeuvres oratoires de maîtres parisiens au 12e siècle: Etude historique et doctrinale*, 2 vols. (Études Augustiniennes, 1975); Ronald James Stansbury, ‘Preaching before the Friars: The Sermons of Ralph Ardent (c. 1130-c. 1215)’ (PhD diss., Ohio State University, 2001).

^11^ All references to the *Vita* that follow have been verified in its manuscript. A full reproduction is available in: Antonio Viñayo González, *Santo Martino de León: Vida y obras narradas por el Tudense* (Isidoriana Editorial, 1984). On Lucas and his works, see Patrick Henriet, ‘“Sanctissima patria”. Points communs entre les trois oeuvres de Lucas de Tuy’, *Cahiers de linguistique et de civilisation hispaniques médiévales* 24 (2001): 249–78; Henriet, ‘Xénophobie et intégration à Léon au XIIIe siècle. Le discours de Lucas de Túy sur les étrangers’, in *L'étranger au Moyen Âge* (Publications de la Sorbonne, 2000), 37–58.

^12^ On Martin’s sources, see de las Heras, ‘Contra Judaeos’, 156–61; de las Heras, ‘Culture scolaire’, 1–25; Laureano Robles Carcedo, ‘Fuentes del Pensamiento Teologico de Santo Martino’, in *Santo Martino de León* (Isidoriana Editorial, 1987), 601–8. On the *Glossa’s* dissemination, see Lesley Janette Smith, *The Glossa Ordinaria: The Making of a Medieval Bible Commentary* (Brill, 2009), 153–80; and Christopher de Hamel, *Glossed Books of the Bible and the Origins of the Paris Book Trade* (Boydell & Brewer, 1984), 1–13, 49, 89–105, where Iberia remains absent save for two manuscripts in Vic cathedral (thirteenth century).

^13^ On this issue, see Mark J. Clark, *The Making of the Historia Scholastica, 1150–1200* (Pontifical Institute of Mediaeval Studies, 2015), 86–100.

^14^ See Matthew Anthony Doyle, *Peter Lombard and His Students* (Pontifical Institute of Mediaeval Studies, 2016), 81–112.

^15^ ‘Legite etiam, charissimi, inter caeteras sanctarum Scripturarum paginas ad eruditionem vestram hunc librum, non quasi a me editum; sed quasi floribus Novi et Veteris Testamenti compositum. Non ego illum dictavi, sed sanctorum Patrum in eo dicta compilavi’ (PL 208: 31). The same is noted twice in: *Vita Martini*, PL 208: 11, 17.

^16^ See Alexander Marx, ‘Divergent Voices in the Preaching of the Third Crusade: Martin of León’s Reading of the Fall of Jerusalem’, *Crusades* 23 (2024): 25–43 (31–33), discussing how he adapts elements from the *Glossa* (originally penned by Jerome), using these to explain Jerusalem’s loss in 1187.

^17^ The fact that the *Vita* does not speak of a ‘crusade’ is not surprising. As already discussed, the Middle Ages did not know a crusade-specific terminology, primarily understanding crusading as an act of pious pilgrimage – perfectly concurring with the *Vita’s* depiction.

^18^ The *Vita* also notes that he was welcomed by León’s bishop, Manrique de Lara (1181–1205); this defines a timeframe (PL 208: 15). His pilgrimage at Urban III’s time delivers a *terminus post quem*, plus thereafter two years in the Holy Land and the way back to León. As a result, one arrives at the early 1190s.

^19^ See also Lucas of Tuy, *Chronicon mundi*, ed. Emma Falque Rey, CCCM 74: 7: ‘Quid de uenerabili Martino Legionensi presbytero sentiendum, qui tantam a Domino precibus in diuinis Scripturis prerogatiuam obtinuit, ut in expositione Scripturarum sanctissimis primis doctoribus merito ualeat adequari?’

^20^ See Peter A. Linehan, ‘Santo Martino and the Context of Sanctity in 13th-century León’, in *Santo Martino de León* (Isidoriana Editorial, 1987), 689–97 (697).

^21^ Henriet, ‘Sanctissima patria’, 257–61; Henriet, ‘Un exemple de religiosité politique: saint Isidore et les rois de León (XIe-XIIIe siècle)’, in *Fonctions sociales et politiques du culte des saints dans les sociétés de rite grec et latin au Moyen Âge et à l'époque moderne*, ed. Marek Derwich and Mikhaïl Vladimirovich Dmitriev (Larhcor, 1999), 77–95 (84–85).

^22^ See, e.g., Helen Birkett, ‘Constructing the Text: A Comparative Study of Two Saints' Lives Written c. 1200’, in *Hagiography and the History of Latin Christendom, 500–1500*, ed. Samantha Kahn Herrick (Brill, 2020), 13–32 (17, 22).

^23^ ‘Episcopi quoque et barones regni eum nimio venerabantur affectu, et ejus sacris monitis et praeceptis se devotissime submittebant.’ (PL 208: 16–17).

^24^ See de las Heras, ‘Contra Judaeos’, 151–5.

^25^ See Nicole Bériou, *L'avènement des maîtres de la Parole. La prédication à Paris au XIIIe siècle*, 2 vols. (Institut d'Études Augustiniennes, 1998), 1: 108; Louis-Jacques Bataillon, ‘Sermons rédigés, sermons réportés (XIIIe siècle)’, in *La prédication au XIIIe siècle en France et Italie* (Variorum, 1993), 69–86 (75–77).

^26^ See Antonio Viñayo González, *Abecedario-Bestiario de los Códices de Santo Martino* (Isidoriana Editorial, 1986), 27–34; Amélie de las Heras, ‘Le livre de l'Apocalypse chez Martin de León (m. 1203), entre commentaire et sermon. Une “lectio divina” tournée vers l'action’, *Mélanges de la Casa de Velázquez* 49 (2019), 61–84 (68–69).

^27^ *Liber Sermonum, Praefatio*, PL 208: 27; Peter Lombard, *Sententiae, Prologus*, PL 192: 522. On these developments, see Mary A. Rouse, ‘“Statim invenire”: Schools, Preachers, and New Attitudes to the Page’, in *Authentic Witnesses: Approaches to Medieval Texts and Manuscripts* (University of Notre Dame Press, 1991), 191–219.

^28^ *Vita Martini*, PL 208: 17: ‘Regina vero Berengaria, ut comperit desiderium sancti viri, sufficientes expensas praebuit, ex quibus vir sanctus sua peregit volumina […]’.

^29^ See Bériou, *L'avènement*, 1: 91.

^30^ All titles and other paratextual elements are recorded in the appendix as they appear in the manuscript.

^31^ Rucquoi, ‘L’invective’, 135–51; Antonio Viñayo González, *San Martín de León y su Apologética antijudía* (Consejo Superior de Investigaciones Científicas, 1948).

^32^ See, e.g., several contributions in: *The Sermon*, ed. Beverly Mayne Kienzle (Brepols, 2000).

^33^ Plausibly, the codex originated after 1197 (as indicated by the fact that the Leonese queen financed it), but Martin started ordering his notes and making plans for an opus magnum from 1185 onwards.

^34^ See Alexander Marx, ‘Jerusalem as the Travelling City of God: Henry of Albano and the Preaching of the Third Crusade’, *Crusades* 20 (2021): 83–120.

^35^ Petrus Cantor, *Verbum Abbreviatum*, PL 205: 25; discussed by Riccardo Quinto, ‘Peter the Chanter and the “Miscellanea del Codice del Tesoro” (Etymology as a Way for Constructing a Sermon)’, in *Constructing the Medieval Sermon*, ed. Roger Andersson (Brepols, 2007), 33–81 (68–70).

^36^ See Caroline Walker Bynum, ‘The Spirituality of Regular Canons in the Twelfth Century’, in *Jesus as Mother: Studies in the Spirituality of the High Middle Ages* (University of California Press, 1982), 22–58, esp. 36–40, 53–58.

^37^ See Franco Morenzoni, *Des écoles aux paroisses. Thomas de Chobham et la promotion de la prédication au début du XIIIe siècle* (Institut d'Études Augustiniennes, 1995).

^38^ ‘[…] antichristi praesumptio et calliditas confunditur; generalis omnium resurrectio praedicatur; judicium futurum in fine mundi praeostenditur […]’ (PL 208: 32).

^39^ See, e.g., Jay Rubenstein, ‘Crusade and Apocalypse: History and the Last Days’, *Quaestiones medii aevi novae* 21 (2016): 159–88.

^40^ See Mariel Pérez, ‘Canónigos regulares y redes parroquiales en la diócesis de León (siglos XII-XIII)’, *Hispania sacra* 75 (2023): 307–21.

^41^ On this idea, see Rouse, ‘Statim invenire’, 205–6.

^42^ See esp. Jessalynn L. Bird, ‘“Theologians know best”: Paris-trained Crusade Preachers as Mediators Between Papal, Popular and Learned Crusading Pieties’, *Journal of Medieval History* 49 (2023): 1–19.

^43^ See, e.g., Christoph T. Maier, ‘Papal Crusade Propaganda and Attacks Against Jews in France in the 1230s: A Breakdown of Communication?’, *Journal of Medieval History* 49 (2023): 339–52.

^44^ See Joseph F. O'Callaghan, *Reconquest and Crusade in Medieval Spain* (University of Pennsylvania Press, 2003), 50–76; Carlos de Ayala Martínez, ‘Holy War and Crusade during the Reign of Alfonso VIII’, in *King Alfonso VIII of Castile: Government, Family, and War*, ed. Miguel Gómez and Kyle C. Lincoln (Fordham University Press, 2019), 118–42 (130–34).

^45^ See Marx, *Early University*; Gaposchkin, *Weapons*, 77–78; Christopher Matthew Phillips, ‘The Typology of the Cross and Crusade Preaching’, in *Crusading in Art, Thought, and Will*, ed. Matthew Parker and Ben Halliburton (Brill, 2018), 166–85 (174–81).

^46^ See Jessalynn L. Bird, ‘Paris Masters and the Justification of the Albigensian Crusade’, *Crusades* 6 (2007): 117–55; Beverly Mayne Kienzle, *Cistercians, Heresy and Crusade in Occitania, 1145 - 1229: Preaching in the Lord's Vineyard* (Boydell & Brewer, 2001).

^47^ See Stephen C. Ferruolo, *The Origins of the University: The Schools of Paris and Their Critics, 1100–1215* (Stanford University Press, 1987); Johannes M. M. H. Thijssen, ‘Master Amalric and the Amalricians: Inquisitorial Procedure and the Suppression of Heresy at the University of Paris’, *Speculum* 71 (1996): 43–65.

^48^ Jeremy Cohen, *Living Letters of the Law: Ideas of the Jew in Medieval Christianity* (University of California Press, 1999), 2–3. The same has been proposed for Martin’s work and its discussions of Jews in de las Heras, ‘Contra Judaeos’, 162–70.

^49^ Bird, ‘Crusade and Reform’; Baldwin, *Masters*; Philippe Buc, *L’Ambiguité du Livre. Prince, Pouvoir, et Peuple dans les Commentaires de la Bible au Moyen Age* (Beauchesne, 1994).

^50^ Gaposchkin, *Weapons*, esp. 32–33; Katherine Allen Smith, *The Bible and Crusade Narrative in the Twelfth Century* (Boydell & Brewer, 2020), 15–47. See also Sylvia Schein, *Gateway to the Heavenly City: Crusader Jerusalem and the Catholic West (1099–1187)* (Routledge, 2005).

^51^ See Kristin Skottki, ‘“Until the Full Number of Gentiles Has Come In”: Exegesis and Prophecy in St Bernard’s Crusade-Related Writings’, in *The Uses of the Bible in Crusader Sources*, ed. Elizabeth Lapina and Nicholas Morton (Brill, 2017), 237–72 (252).

^52^ Cohen, *Living Letters*, 156–65.

^53^ See esp. David d'Avray, *The Preaching of the Friars: Sermons Diffused from Paris Before 1300* (Clarendon Press, 1985), 104–31.

^54^ On Martin’s anti-Judaism, see also de las Heras, ‘Contra Judaeos’, 147–74; Rucquoi, ‘L’invective’, 135–51.

^55^ See, e.g., *Liber Sermonum, Sermo 18*, PL 208: 811–12; *Sermo 22*, PL 208: 843; *Sermo 23*, PL 208: 907–8; *Sermo 29*, PL 208: 1050–51. For such concerns in León, see Julie A. Harris, ‘Jews, Real and Imagined, at San Isidoro de León and Beyond’, *Medieval Encounters* 25 (2019): 180–201 (187–89); Patrick S. Marschner, ‘Biblical Elements and the ‘Other’ in the Chronicon regum Legionensium’, *Medieval Worlds* 8 (2018): 66–85 (80).

^56^ See Marx, ‘Divergent Voices’, 38–41; Marx, *Early University*, 196–214. Martin often speaks directly to his audience (second person plural) while calling them Jews. Yet, numerous occasions make apparent that he is addressing Christians, for example, he offers them the option of doing penance (PL 208: 862).

^57^ *Vita Martini*, PL 208: 20. See also Lucas of Tuy, *Chronicon*, 326, 331; and Harris, ‘Jews’, 182.

^58^ ‘Qui respondens, dixit eis: Constantes estote, ne formidetis; pro certo scientes, quoniam haec civitas ab his regibus non capietur, quoniam Dominus protegit eam precibus beati Isidori confessoris. Scitote etiam quoniam in brevi rex Castellae recipiet nuntium, quod rex Legionis cum exercitu magno invadit regnum ejus; et recedens ab obsidione istius urbis parabit obviam ei, sed nullus erit inter eos armorum conflictus [end of speech]. Ut vir Dei praedixit, sic omnino accidit, et manifestatus est dare in eo spiritus prophetae’ (PL 208: 20).

^59^ See Rucquoi, ‘L’invective’, 138, 150. For discussions of Jews in León, encouraging such a view, see Harris, ‘Jews’, 180–83; Viñayo González, *Apologética antijudia*, 63–76. On reviewing *convivencia*, see, e.g., Alex James Novikoff, ‘Between Tolerance and Intolerance in Medieval Spain: An Historiographic Enigma’, *Medieval Encounters* 11 (2005): 7–36; Christophe Cailleaux, ‘Chrétiens, juifs et musulmans dans l'Espagne médiévale. La convivencia et autres mythes historiographiques’, *Cahiers de la mediterranée* 86 (2013): 257–71.

^60^ Isidore of Seville, *Etymologiae* (8.5), PL 82: 298–305. I leave it to other scholars to examine how Martin updated Isidore’s ideas in light of contemporary circumstances.

^61^ The only part that cannot be aligned with the two treatises is the first section (PL 208: 83–106), which I have not investigated, but as the appendix shows, crusade-related subjects do not play any role therein.

^62^ See, e.g., John V. Tolan, *Saracens: Islam in the Medieval European Imagination* (Columbia University Press, 2002), 135–69, for a discussion of the twelfth-century trend of labelling Islam as a heresy. For other cases of entangling the groups, see, e.g., *Liber Sermonum, Sermo 18*, PL 208: 818; *Sermo 22*, PL 208: 864; *Sermo 23*, PL 208: 871, 886, 894; *Expositio libri Apocalypsis*, PL 209: 310, 347, 384.

^63^ Though the philosophers are absent as the subject of a dedicated book, the work reproaches them on several occasions. On Alan, see Tolan, *Saracens*, 165–66; Gillian Rosemary Evans, *Alan of Lille: The Frontiers of Theology in the Later Twelfth Century* (Cambridge University Press, 1983), 10–11, 116–32.

^64^ Lucas even refers occasionally to Martin’s texts. See Lucas of Tuy, *De altera vita*, ed. Emma Falque Rey, CCCM 74A: xxxii, 25, 119, using *Liber Sermonum, Sermo 4*, PL 208: 313, 493. See also Henriet, ‘Sanctissima patria’, 272; Henriet, ‘Hagiographie léonaise et pédagogie de la foi: les miracles d'Isidore de Séville et la lutte contre l'hérésie (XIe-XIIIe siècle)’, in *L'enseignement religieux dans la Couronne de Castille*, ed. Daniel Baloup (Casa de Velázquez, 2003), 1–28.

^65^ *Papsturkunden in Portugal*, ed. Carl Erdmann (Weidmann, 1927), 376–77; discussed by Miguel Gómez and Kyle C. Lincoln, ‘“The Sins of the Sons of Men”: A New Letter of Pope Celestine III concerning the 1195 Crusade of Alarcos’, *Crusades* 16 (2017): 55–63 (61). See also O'Callaghan, *Reconquest*, 62–64.

^66^ See Peter A. Linehan, *History and the Historians of Medieval Spain* (Clarendon Press, 1993), 253, 258.

^67^ See also Lucas of Tuy, *Chronicon*, 326, which also relates the *Liber’s* composition to these conflicts.

^68^ See Ayala Martínez, ‘Holy War’, 118–42. On the active role of the queen, see Bianchini, *Queen's Hand*, 37–68.

^69^ See Ferruolo, *Origins*, 222–77; Baldwin, *Masters*, 1: 76–82, 97–107.

^70^ Bernard of Clairvaux, *De consideratione* (5.6–7), in *Sämtliche Werke*, ed. and tr. Gerhard B. Winkler, 10 vols. (Tyrolia-Verlag, 1990), 1: 794–802. On this effort, see Ferruolo, *Origins*, 61–64.

^71^ One also gets 199 hits for *infidel**, 229 hits for *incredul** (thus straddling the nouns and not only the groups), and 237 hits for *impi** (reduced by hits for *impingere*, the only overlapping word, if I am not mistaken).

^72^ Tolan, *Saracens*, xix, 126–28; see also Skottki, ‘Number’, 267–70.

^73^ See Robin J. Vose, *Dominicans, Muslims, and Jews in the Medieval Crown of Aragon* (Cambridge University Press, 2009), 27; Matthias M. Tischler, ‘Warum Clunys Islamprojekt zunächst scheitern musste und schließlich doch ein Erfolg wurde. Die Auseinandersetzung des Reformmönchtums mit dem Islam vor und nach Petrus Venerabilis (11.-13. Jahrhundert)’, in *Die Wirkmacht klösterlichen Lebens. Modelle – Ordnungen – Kompetenzen – Konzepte*, ed. Mirko Breitenstein and Gert Melville (Schnell und Steiner, 2020), 275–94 (291).

^74^ On the first, see Pamela Anne Patton, ‘Demons and Diversity in León’, *Medieval Encounters* 25 (2019): 150–79. On the second, see Marschner, ‘Biblical Elements’, 66–85; Simon Barton, ‘Islam and the West: A View from Twelfth-Century Leon’, in *Cross, Crescent and Conversion: Studies on Medieval Spain and Christendom*, ed. Simon Barton and Peter A. Linehan (Brill, 2008), 153–74 (158–64).

^75^ Smith, *Crusade Narrative*, 128–36; and Richard W. Southern, *Western Views of Islam in the Middle Ages* (Harvard University Press, 1962), 25–28. See also Nicholas Morton, *Encountering Islam on the First Crusade* (Cambridge University Press, 2016), 273–75.

^76^ See Patrick S. Marschner, *Das neue Volk Gottes in Hispanien: Die Bibel in der christlich-iberischen Historiographie vom 8. bis zum 12. Jahrhundert* (LIT-Verlag, 2023), 102–6, 467–97; Hélène Sirantoine, ‘What's in a Word? Naming “Muslims” in Medieval Christian Iberia’, in *Making the Medieval Relevant: How Medievalists Are Revolutionising the Present*, ed. Chris Jones, Conor Kostick, and Klaus Oschema (De Gruyter, 2020), 225–38.

^77^ One gets 273 hits for *Jerusalem*; 413 hits for *Israel*; and 98 hits for *Zion*. References to this region and biblical stories located in the same appear throughout the work. However, distribution is still uneven; see the appendix.

^78^ *Liber Sermonum, Sermo 18*, PL 208: 822; *Sermo 22*, PL 208: 843–44; *De sanctis, Sermo 5*, PL 209: 29–38. See Marx, ‘Divergent Voices’, 31–3. On other sermons *De sancta cruce*, see Marx, *Early University*, 173–74, 222–28, also explaining the methodological tools for relating such texts to crusading.

^79^ *Expositio libri Apocalypsis*, PL 209: 359–60. See Marx, *Early University*, 368–407; Jean Flori, *L’islam et la fin des temps. L’interprétation prophétique des invasions musulmanes dans la chrétienté médiévale* (Édition du Seuil, 2007), 302–12. On Martin’s commentary, see also de las Heras, ‘Livre’, 61–84.

^80^ See, e.g., *Liber Sermonum, Sermo 18*, PL 208: 818–20; *Sermo 22*, PL 208: 844, 864; *Sermo 28*, PL 208: 1026. Whereas *indulgentia peccatorum* appears on several occasions, *plena indulgentia* is found twice: *Liber Sermonum, Sermo 4*, PL 208: 126; *Sermo 22*, PL 208: 862. On this subject, see Anne Lise Bysted, *The Crusade Indulgence: Spiritual Rewards and the Theology of the Crusades, c. 1095–1216* (Brill, 2015).

^81^ This draws on Marx, *Early University*, which effectively anchored many sermons texts in the mobilisation of the Third Crusade.

^82^ Jessalynn L. Bird, ‘Preaching the Crusades and the Liturgical Year: The Palm Sunday Sermons’, *Essays in Medieval Studies* 30 (2014): 11–36.

^83^ See Alexander Marx, ‘Constructing and Denying the Enemy: Cistercian Approaches to Preaching the Third Crusade (1187–1192)’, *Cîteaux* 70 (2019): 47–68.

^84^ God’s *virga* is an apocalyptic motif. See, e.g., Is. 11: 4; Rev. 2: 27; 12: 5; 19: 15. On the Corpus Christi, see Henri de Lubac, *Corpus mysticum. L’eucharistie et l’église au moyen âge* (Aubier, 1949); Ernst Kantorowicz, *The King's Two Bodies: A Study in Medieval Political Theology* (Princeton University Press, 1957), 194–206.

^85^ See Marx, ‘Jerusalem’, 106–7; Marx, ‘The Passio Raginaldi of Peter of Blois. Martyrdom and Eschatology in the Preaching of the Third Crusade’, *Viator* 50 (2019): 197–232 (209–11).

^86^ See Marx, ‘Passio Raginaldi’, 209, 225; Jessalynn L. Bird, ‘James of Vitry's Sermons to Pilgrims’, *Essays in Medieval Studies* 25 (2008): 81–113 (86–87, 101–12).

^87^ ‘Easdem passiones quotidie Ecclesia patitur, quae est de carne ejus, et de ossibus ejus, ut compleat ea quae desunt Christi passionum [Col. 1: 24]. Inde dicit: Aedificavit in gyro meo, et circumdedit me felle et labore [Lam. 3: 5]. Ecclesia enim omnibus obsessa malis, inter scorpiones habitat, ubi sedes est Satanae [Rev. 2: 13]; haereticorum vallatur exercitu; a falsis fratribus quasi felle, et labore potatur; persecutorum undique opprimitur tormentis […]’ (PL 208: 820).

^88^ ‘Sequitur: Ursus insidians factus est mihi; leo in abscondito [Lam. 3: 10]. Historice: Quid terribilius quam auctorem nostrae salutis ursum et leonem fieri nobis? Ursus fera fraudulenta in brachiis et lumbis fortissima. In Nabuchodonosor, et in Vespasiano, et Tito, qui alibi aper et singularis dicitur [Ps. 80: 14], Dominus ursus et leo vocatur, in quibus Dei potestas inimicos ulciscitur. Plangitur ergo juste, quod Deus pius et clemens Dominus, peccatis exigentibus, leo fit vel ursus, ut ejus potentia hostes obsideat, et affligat. Quasi signum ad sagittam [Lam. 3: 12] Hierusalem ponitur, quia circumfusis hostibus undique obsidetur, ut frequenter quasi sagittis vulnerata ad medicum redeat vel coacta. Has sagittas, id est has gentes quasi arcu tenso moderato judicio contra Hierusalem misit, ut vel sic Deum timere disceret, ne hostium suorum sagittas vulnerata deficeret.’ (PL 208: 822).

^89^ See Schein, *Gateway*, 170–74; Marx, *Early University*, 341–67.

^90^ I am currently working on a large project on the reception of the Roman conquest in Latin texts between c.600 and c.1300, and what meaningful historical purposes this event served; this will become my second book. https://www.oeaw.ac.at/imafo/forschung/historische-identitaetsforschung/projekte/weitere-projekte/conquest-of-jerusalem (accessed 09 May 2025).

^91^ ‘His duabus bestiis omnis saevitia diaboli demonstratur; et per leonem ipse diabolus, per ursum vero membra ejus, haeretici videlicet et falsi fratres.’ (PL 208: 823).

^92^ ‘Hierusalem scilicet, quam supra propheta plangebat, jam fortassis in se reversa, imo Christi sponsa sanguine ejus dotata gemit se desolatam, et quasi adulteram a sponso derelictam.’ (PL 208: 823).

^93^ See Marx, *Early University*, 207–14. For sources see, e.g., London, British Library, MS Add 19767, fol. 73v; Peter of Blois, *Conquestio de dilatione vie ierosolimitane*, ed. Robert B. C. Huygens, CCCM 194: 77. It is also present in the crusade encyclical: Thomas W. Smith, ‘Audita Tremendi and the Call for the Third Crusade Reconsidered, 1187–1188’, *Viator* 49 (2018): 63–101 (94–95).

^94^ See Marx, *Early University*, 368–407; Marx, ‘Jerusalem’, 102–10; Flori, *L’islam*, 300–35.

^95^ See Marx, *Early University*, 315–18. For sources see, e.g., Alan of Lille, *Contra Haereticos* (1.20), PL 210: 322; Paris, Bibliothèque de l’Arsenal, MS 543, fols. 207v, 219v; and later: Humbert of Romans, *De predicatione crucis*, ed. Valentin Portnykh, CCCM 279: 59, 82, 118.

^96^ See Paris, Bibliothèque Nationale de France, MS lat. 14859, fol. 233v; discussed by Beverly Mayne Kienzle, ‘Preaching the Cross: Liturgy and Crusade Propaganda’, in *Preaching and Political Society. From Late Antiquity to the End of the Middle Ages*, ed. Franco Morenzoni (Brepols, 2013), 11–46 (23–25).

^97^ The passages discussed in this and the following case study have been verified in the manuscript; I have not found any meaningful deviations between it and the edition.

^98^ See William J. Purkis, *Crusading Spirituality in the Holy Land and Iberia, c. 1095 – c. 1187* (Boydell & Brewer, 2008), 47–57, esp. 51. See also Tolan, *Saracens*, 111–12.

^99^ ‘Erit unum nomen Domini [Zach. 14: 9], prava religione idolorum calcata. Tunc revertetur omnis terra [Zach. 14: 10] in qua habitaverunt Judaei usque ad desertum.’ (PL 209: 150).

^100^ See Flori, *L’islam*, esp. 116–21, 320–25; Tolan, *Saracens*, 40–57. On prophetic fulfilments in the crusades, see Matthew Gabriele, ‘From Prophecy to Apocalypse: The Verb Tenses of Jerusalem in Robert the Monk's Historia of the First Crusade’, *Journal of Medieval History* 42 (2016): 304–16; Matthieu Rajohnson, *L'Occident au regret de Jérusalem (1187-fin du XIVe siècle)* (Classiques Garnier, 2021), 213–17.

^101^ ‘Inimici Ecclesiae sunt daemones, haeretici, et falsi Christiani, et persecutores pagani. Nam et Judaei graviter eam afflixerunt in ipso capite suo Christo, et suis membris […]’ (PL 209: 161).

^102^ See Marx, *Early University*, 177–96; Phillips, ‘Typology’, 174–81. For sources see, e.g., Peter of Blois, *Conquestio*, 85–86; Henry of Albano, *Ep.31*, PL 204: 247; Innocent III, *De diversis, Sermo 6*, PL 217: 677.

^103^ On Mt. 16: 24 and the crusades, see Gaposchkin, *Weapons*, 66; Purkis, *Spirituality*, 30–47, 89–93.

^104^ On martyrdom in the crusading context, see Miikka Tamminen, *Crusade Preaching and the Ideal Crusader* (Brepols, 2018), 169–201; Philippe Buc, *Holy War, Martyrdom, and Terror: Christianity, Violence, and the West, ca. 70 C.E. to the Iraq War* (University of Pennsylvania Press, 2015), 84–85, 167–76.

^105^ Beyond cases already addressed, see *Liber Sermonum, Sermo 22*, PL 208: 850, 856; *Sermo 27*, PL 208: 998–99.

^106^ See Gaposchkin, *Weapons*, 193–94, 208–19.

^107^ See Jessalynn L. Bird, ‘Rogations, Litanies, and Crusade Preaching: The Liturgical Front in the Late Twelfth and Early Thirteenth Centuries’, in *Papacy, Crusade, and Christian-Muslim Relations*, ed. Jessalynn L. Bird (Amsterdam University Press, 2018), 155–93. On blending liturgy and crusading, see also Cecilia Gaposchkin, ‘The Echoes of Victory: Liturgical and Paraliturgical Commemorations of the Capture of Jerusalem in the West’, *Journal of Medieval History* 40 (2014): 237–59. On militant liturgy in the Peninsula, see O'Callaghan, *Reconquest*, 177–208; Manuel Rojas Gabriel, ‘On the Path of Battle. Divine Invocations and Religious Liturgies Before Pitched Battles in Medieval Iberia (c. 1212-c. 1340): An Introduction’, in *Crusading on the Edge. Ideas and Practice of Crusading in Iberia and the Baltic Region*, ed. Torben K. Nielsen and Iben Fonnesberg-Schmidt (Brepols, 2016), 275–97.

^108^ On this nexus, see also Gaposchkin, *Weapons*, 57, 61–62; Buc, *Holy War*, 78–79.

^109^ On Nineveh in crusade-related sermons, see Bird, ‘Rogations’, 158, 170; Bird, ‘Damietta the Whore, the Purification of the Virgin Mary, and the Crusade Movement’, *Medieval Sermon Studies* 65 (2021): 3–25 (14).

^110^ See Gaposchkin, *Weapons*, 130–64.

^111^ See Marx, ‘Jerusalem’, 96–102; Rajohnson, *L'Occident*, 223–48.

^112^ ‘Quando rectores populi recta via non gradiuntur, graviter corruunt, qui sequuntur. […] Ecclesia ergo, peccatis tam rectorum quam subditorum exigentibus amaritudine opprimitur; quia saepe ei fames incumbit, pagana gens illius terminos invadit, luporum rabies humana membra laniare praesumit, haereticorum perversitas ejus filios seducere ac sui erroris veneno inficere appetit.’ (PL 208: 1063).

^113^ On God using human agents, see Marx, ‘Enemy’, 47–68; Keagan Brewer, ‘God’s Devils: Pragmatic Theodicy in Christian Responses to Ṣalāḥ al-Dīn’s Conquest of Jerusalem in 1187’, *Medieval Encounters* 27 (2021): 125–64.

^114^ See Skottki, ‘Number’, 257–60; Hans-Dietrich Kahl, ‘“ … Auszujäten von der Erde die Feinde des Christentums … ” Der Plan zum “Wendenkreuzzug” von 1147 als Umsetzung sibyllinischer Eschatologie’, in *Heidenfrage und Slawenfrage im deutschen Mittelalter* (Brill, 2011), 633–66.

^115^ On such ideas, see Christian Hofreiter, *Making Sense of Old Testament Genocide: Christian Interpretations of Herem Passages* (Oxford University Press, 2018), esp. 167–93, 247–51. The blurry labelling as ‘pagans’, instead of ethnic denominations, concurs with Bernard’s writings on the matter (see Kahl, ‘Auszujäten’, 634–36).

^116^ *Papsturkunden in Spanien*, ed. Daniel Berger, Klaus Herbers, and Thorsten Schlauwitz (De Gruyter, 2020), no. CCLIII, 466–68; and no. CCLVI, 474–77. See also O'Callaghan, *Reconquest*, 57–58; Ayala Martínez, ‘Holy War’, 126.

^117^ See Purkis, *Spirituality*, 131–37; Patrick J. O'Banion, ‘What has Iberia to do with Jerusalem? Crusade and the Spanish Route to the Holy Land in the Twelfth Century’, *Journal of Medieval History* 34 (2008): 383–95; Nikolas Jaspert, ‘Eroberung – Rückeroberung – Glaubenskampf – Gotteskrieg. Die Levante und die Iberische Halbinsel im Vergleich’, in *Herrschaft über fremde Völker und Reiche. Formen, Ziele und Probleme der Eroberungspolitik im Mittelalter*, ed. Hermann Kamp (Jan Thorbecke, 2022), 249–89 (271–73).

^118^ I searched the *Liber* for details that would relate to the Peninsula such as *legio* (León). *Hispania* only appears when referring to Isidore as the *doctor hispaniarum*. This stands in sharp contrast to Lucas of Tuy’s works stressing the role of *hispania*. See Henriet, ‘Sanctissima patria’, 257–64; see also Hélène Sirantoine, *Imperator hispaniae: Les idéologies impériales dans le royaume de León (IXe-XIIe siècles)* (Casa de Velázquez, 2013).

^119^ Nikolas Jaspert, ‘Eleventh-Century Pilgrimage from Catalonia to Jerusalem: New Sources on the Foundations of the First Crusade’, *Crusades* 14 (2015): 1–47; see also Purkis, *Spirituality*, 165–76. On warfare within the Peninsula, see Marschner, *Volk Gottes*; Alexander Pierre Bronisch, *Reconquista und Heiliger Krieg. Die Deutung des Krieges im christlichen Spanien von den Westgoten bis ins frühe 12. Jahrhundert* (Aschendorff, 1998).

^120^ See Gómez and Lincoln, ‘Sins’, 55–63; Matthias Maser, ‘“Dissolve colligationes impietatis” – Papst Innozenz III. und die Anfänge seiner Politik des negotium crucis auf der Iberischen Halbinsel (1198–1204)’, in *Europa 1215. Politik, Kultur und Literatur zur Zeit des IV. Laterankonzils*, ed. Michele Camillo Ferrari and Klaus Herbers (Böhlau, 2018), 123–52 (129–38).

^121^ *De diversis, Sermo 9*–*10*, PL 209: 125–38 (125, 134). He also included two sermons on the translation of Isidore’s remains to León: *De sanctis, Sermo 8*–*9*, PL 209: 51–62.

^122^ See Marx, ‘Enemy’, 47–68.

^123^ See Jaspert, ‘Eroberung’, 260–61, 269–70; Ayala Martínez, ‘Holy War’, 128; Patrick S. Marschner, ‘The Chronicle of Sampiro, the Arabs, and the Bible: Eleventh-Century Christian-Iberian Strategies of Identifying the Cultural and Religious “Other”’, in *Transcultural Approaches to the Bible: Exegesis and Historical Writing across Medieval Worlds*, ed. Matthias M. Tischler and Patrick S. Marschner (Brepols, 2021), 75–105 (84–93).

^124^ For such a strategy in other texts, see Tolan, *Saracens*, 124.

^125^ *Liber Sermonum, Sermo 4*, PL 208: 164–67. A key passage reads: ‘Aliquando vero Aethiopiae nomine specialiter gentilitas designari solet infidelitatis prius nigra peccatis’ (164). See also *Expositio libri Apocalypsis*, PL 209: 335, where Revelation’s *equus niger* is identified with heretics and pagans (*gentilis populus*); and see *Liber Sermonum, Sermo 29*, PL 208: 1050: ‘Aethiopicum colorem, id est vitiorum nigredinem, per puram confessionem et dignam poenitentiam deponamus’. Such ideas are also found in other Leonese sources (see Patton, ‘Demons’, 150–79).

^126^ See Kyle C. Lincoln, ‘Riots, Reluctance, and Reformers: The Church in the Kingdom of Castile and the IV Lateran Council’, *Journal of Medieval Iberian Studies* 12 (2020): 230–47; Jessalynn L. Bird, ‘Inquisitorial Identity and Authority in Thirteenth-Century Exegesis and Sermons: Jean Halgrin d'Abbeville, Jacques de Vitry and Humbert of Romans’, in *Inquisition and Knowledge, 1200–1700*, ed. Peter Biller and Lucy J. Sackville (York Medieval Press, 2022), 37–56.

^127^ See Damian J. Smith, *Crusade, Heresy, and Inquisition in the Lands of the Crown of Aragon (c. 1167–1276)* (Brill, 2010), 36. See also Maser, ‘Innozenz III.’, 126–29, 138–49, discussing Innocent’s concerns before Las Navas, related to sin and the Peninsula (including Alfonso IX’s incestuous marriage).

## Appendix: Composition of the Liber Sermonum, León, San Isidoro, MS 11

The table presents the work’s composition faithful to its only surviving manuscript, which includes all of Martin’s extant writings, and which consists of two codices, both preceded by the same prologue. It catalogues the occurrences of words designating those groups that are presented as targets in the work’s prologue, including the various terms for ‘pagans’ (especially *pagani* and *gentiles*, but also the motif of *idola*). This is based on surveys conducted in the digitized versions of the *Patrologia Latina*. The table also records how exactly I searched there, for example, via ‘haereti*’. The column on the right notes other crusade elements found in these texts: ‘Jerusalem’, the ‘Holy Land’ (primarily straddling the terms *Israel* and *Zion*), ‘penance’, ‘martyrdom’, and militant imagery (mainly the terms *arma* and *gladius*). ‘None’ means that all crusade elements remain <1; ‘Minor’ that they all remain <5. The normal font indicates 5 + and bold 10 + hits. Exceptionally high numbers are noted within brackets. The results need to be considered in conjunction with a text’s length, since this varies greatly. The numbering of the sermons (including the sections within *Sermo 4*) follows the *Patrologia Latina*, in order to make the texts identifiable in the edition. The texts’ titles, however, are faithful to the manuscript (which are, in many cases, identical with the edition).

**Table:**

Liber Sermonum, Codex (I)	*philosoph**	*judae**	*haereti**	Pagans	Other terms	Crusade elements
fol. 0v	Preface (*prephatio*) and table of contents (*capitula in libro sermonum*)					
Prologus in libro sermonum fols. 1r – 2r (PL 208:29–31)	1	1	1	*pagan**: 1 *gentil**: 0 *ismael**: 0 *idol**: 1	*infidel**: 0 *incredul**: 0 *impi**: 0 *blasphem**: 0	
Sermo 1 In adventu domini fols. 2r – 4r (PL 208:31–37)	0	2	0	*gentil**: 3 *idol**: 1	*infidel**: 2 *impi**: 1 *blasphem**: 1	Minor
Sermo 2 In adventu domini fols. 4r – 14r (PL 208:37–65)	0	26	2	*gentil**: 3 *idol**: 4	*infidel**: 4 *incredul**: 8 *impi**: 21 *blasphem**: 1	Holy Land; **Penance**
Sermo 3 In natale domini fols. 14r – 20v (PL 208:65–83)	0	15	0	*pagan**: 1 *gentil**: 3 *idol**: 3	*infidel**: 5 *incredul**: 6 *impi**: 2	Minor
Sermo 4 In natale domini fols. 20v – 212v (PL 208:83–550)	Divided into 38 sections (in the edition)					
Section I (PL 208:83–106)	1	1	5	*idol**: 2	*incredul**: 1 *impi**: 3	None
Sections II – XXVIII **Anti-Jewish treatise** (PL 208:106–405)	5	805	20	*pagan**: 12 *gentil**: 50 *ismael**: 10 *idol**: 36	*infidel**: 67 *incredul**: 144 *impi**: 23 *blasphem**: 26	**Jerusalem**; **Holy Land**; **Penance**; **Militant imagery**
Sections XXIX – XXXVIII **Anti-heretical treatise** (PL 208:405–550)	14	78	334	*pagan**: 8 *gentil**: 20 *idol**: 12	*infidel**: 13 *incredul**: 4 *impi**: 32 *blasphem**: 44	**Holy Land**; **Penance**; **Martyrdom**
New foliation starts here						
Sermo 5 In epiphania domini fols. 1r – 2v (PL 208:550–54)	0	23	0	*gentil**: 4 *idol**: 1	None	Minor
Sermo 6 In septuagesima fols. 2v – 4r (PL 208:554–59)	0	1	0	None	*infidel**: 1	None
Sermo 7 In septuagesima fols. 4r – 22v (PL 208:559–607)	2	4	3	*gentil**: 1 *idol**: 1	*incredul**: 2 *impi**: 3	**Jerusalem**; **Holy Land**
Sermo 8 In sexagesima fols. 22v – 28r (PL 208:607–21)	0	1	3	*pagan**: 1	*infidel**: 2 *incredul**: 3 *impi**: 1	Penance; Martyrdom
Sermo 9 In quinquagesima fols. 28r – 32r (PL 208:621–31)	0	4	1	*ismael**: 1	*impi**: 2	Holy Land
Sermo 10 In capite ieiunii fols. 32r – 48v (PL 208:631–75)	0	12	6	*gentil**: 1 *idol**: 2	*infidel**: 3 *incredul**: 1 *impi**: 12 *blasphem**: 2	**Holy Land**; **Penance (161)**; Militant imagery
Sermo 11 In prima dominica quadragesimae fols. 48v – 72r (PL 208:675–733)	1	1	2	*idol**: 3	*infidel**: 1 *incredul**: 1 *impi**: 6 *blasphem**: 2	**Holy Land**; **Penance**; **Militant imagery**
Sermo 12 In quadragesima fols. 72r – 75r (PL 208:733–41)	0	1	0	None	*impi**: 2	Minor
Sermo 13 Dominica secunda in quadragesima fols. 75r – 80v (PL 208:741–55)	0	24	2	*gentil**: 5 *ismael**: 5	*incredul**: 1	Minor
Sermo 14 In dominica tertia quadragesimae fols. 80v – 84v (PL 208:755–69)	0	5	0	*gentil**: 1 *ismael**: 1 *idol**: 15	*blasphem**: 1	Minor
Sermo 15 In dominica quarta quadragesimae fols. 84v – 92r (PL 208:769–91)	1	5	3	*gentil**: 1 *idol**: 3	*infidel**: 4 *impi**: 2	**Holy Land**; **Militant imagery**
Sermo 16 De passione domini fols. 92r – 95r (PL 208:791–99)	0	4	1	*gentil**: 1 *idol**: 1	*impi**: 2 *blasphem**: 1	Jerusalem; **Penance**
Sermo 17 De passione domini fols. 95r – 99v (PL 208:799–811)	0	9	0	*gentil**: 1	*incredul**: 1 *impi**: 1	Holy Land
Sermo 18 In ramis palmarum fols. 99v – 106r (PL 208:811–27)	1	15	6	*gentil**: 1 *idol**: 1	*infidel**: 1 *impi**: 1	Jerusalem; Holy Land
Sermo 19 In ramis palmarum fols. 106r – 108v (PL 208:827–31)	0	3	0	*gentil**: 2	None	Minor
Sermo 20 In ramis palmarum fols. 108v – 110v (PL 208:831–37)	0	5	0	*gentil**: 3 *idol**: 1	None	Minor
Sermo 21 In coena domini fols. 110v – 112v (PL 208:837–43)	0	0	0	None	None	None
Sermo 22 In coena domini fols. 112v – 121v (PL 208:843–65)	0	32	4	*pagan**: 1 *idol**: 2	*infidel**: 3 *incredul**: 8 *impi**: 3 *blasphem**: 2	**Holy Land**; **Penance (24)**; **Militant imagery**
Sermo 23 In coena domini fols. 121v – 144r (PL 208:865–921)	1	145	3	*gentil**: 13 *ismael**: 1 *idol**: 8	*infidel**: 11 *incredul**: 11 *impi**: 26 *blasphem**: 9	**Jerusalem (21)**; **Holy Land**; **Penance**; **Militant imagery**
De diversis, Sermo 1–10 fols. 144r – 162r (PL 209:93–137)	0	11	0	*gentil**: 9 *idol**: 8	*infidel**: 5 *impi**: 3	**Jerusalem**; **Holy Land**; Penance
De sanctis, Sermo 1 fols. 162r – 164r (PL 209:9–13)	0	0	1	None	*impi**: 1	Militant imagery
Sermo 33 In festivitate sancti spiritus fols. 164r – 165v (PL 208:1265–69)	0	3	0	None	*blasphem**: 1	None
De sanctis, Sermo 2–9 fols. 165v – 196r (PL 209:13–93)	4	14	3	*pagan**: 3 *gentil**: 2 *idol**: 7	*infidel**: 1 *incredul**: 2 *impi**: 4	Jerusalem; **Holy Land**; **Penance**; **Militant imagery**
**Total**	**31**	**1250**	**400**	***pagan**: 27** ***gentil**: 124** ***ismael**: 18** ***idol**: 112**	***infidel**: 123** ***incredul**: 193** ***impi**: 151** ***blasphem**: 90**	

Liber Sermonum, Codex (II)	*philosoph**	*judae**	*haereti**	Pagans	Other terms	Crusade elements
fol. 1r	Preface (*prefatio*) and table of contents (*capitula in libro sermonum*)					
Prologus in libro sermonum fols. 1v – 2v (PL 208:29–31)	1	1	1	*pagan**: 1 *gentil**: 0 *ismael**: 0 *idol**: 1	*infidel**: 0 *incredul**: 0 *impi**: 0 *blasphem**: 0	
Sermo 24 De resurrectione domini fols. 2v – 3v (PL 208:921–25)	0	0	0	None	None	None
Sermo 25 De resurrectione domini fols. 3v – 5v (PL 208:925–31)	0	1	0	None	*incredul**: 1 *impi**: 1	None
Sermo 26 De resurrectione domini fols. 5v – 21r (PL 208:931–77)	1	40	0	*gentil**: 11 *idol**: 4	*infidel**: 6 *impi**: 2 *blasphem**: 1	**Jerusalem**; **Holy Land**; Penance; **Militant imagery**
De diversis, Sermo 11 fols. 21r – 38r (PL 209:137–83)	1	45	3	*pagan**: 1 *gentil**: 12 *ismael**: 1 *idol**: 5	*infidel**: 15 *impi**: 1 *blasphem**: 1	**Jerusalem**; **Holy Land**; Penance; **Martyrdom (26)**; **Militant imagery**
Sermo 27 In dominica secunda post pascha fols. 38r – 50v (PL 208:977–1009)	0	10	4	*gentil**: 1 *ismael**: 2 *idol**: 3	*infidel**: 1 *impi**: 3 *blasphem**: 1	**Jerusalem (31)**; **Holy Land**; Penance
Explanatio in epistolam Jacobi apostoli fols. 50v – 62v (PL 209:183–217)	0	4	0	*gentil**: 1 *idol**: 2	*infidel**: 2 *incredul**: 1 *impi**: 5 *blasphem**: 5	Penance
Explanatio in epistolam I Petri apostoli fols. 62v – 75v (PL 209:217–53)	1	8	2	*pagan**: 1 *gentil**: 3 *idol**: 2	*infidel**: 4 *incredul**: 7 *impi**: 4 *blasphem**: 4	Holy Land; Penance
Explanatio in epistolam I Joannis fols. 75v – 94r (PL 209:253–99)	2	7	13	*pagan**: 1 *gentil**: 1 *ismael**: 1 *idol**: 10	*infidel**: 1 *incredul**: 1 *impi**: 3	Minor
Sermo 28 In litaniis fols. 94r – 106v (PL 208:1009–35)	0	2	6	*gentil**: 1	*infidel**: 2 *impi**: 5 *blasphem**: 1	Holy Land; Militant imagery
Sermo 29 In rogationibus fols. 106v – 126r (PL 208:1035–85)	0	7	1	*pagan**: 5 *gentil**: 1 *idol**: 4	*infidel**: 2 *impi**: 2 *blasphem**: 3	**Jerusalem**; **Holy Land**; **Penance (59)**; **Militant imagery**
Sermo 30 In ascensione domini fols. 126r – 171v (PL 208:1085–1197)	1	71	6	*pagan**: 3 *gentil**: 13 *idol**: 1	*infidel**: 17 *incredul**: 12 *impi**: 20 *blasphem**: 3	Minor
Sermo 31 In ascensione domini fols. 171v – 174r (PL 208:1197–1203)	0	1	1	None	*impi**: 2 *blasphem**: 4	Jerusalem
Explanatio libri Apocalypsis fols. 174r – 221r (PL 209:299–420)	3	42	57	*pagan**: 2 *gentil**: 22 *idol**: 18	*infidel**: 13 *incredul**: 3 *impi**: 35 *blasphem**: 23	**Jerusalem**; **Holy Land**; **Penance (42)**; **Martyrdom (21)**; **Militant imagery**
Sermo 32 In festo sancti spiritus fols. 221r – 256v (PL 208:1203–65)	1	15	0	*gentil**: 13 *idol**: 1	*infidel**: 9 *incredul**: 4 *impi**: 2 *blasphem**: 1	Jerusalem; **Holy Land**; **Penance**; Martyrdom
Sermo 34 In festivitate sanctae trinitatis fols. 256v – 292r (PL 208:1269–1350)	3	50	14	*pagan**: 2 *gentil**: 1 *idol**: 2	*infidel**: 4 *incredul**: 7 *impi**: 1	Minor
**Total**	**14**	**304**	**108**	***pagan**: 16** ***gentil**: 80** ***ismael**: 4** ***idol**: 53**	***infidel**: 76** ***incredul**: 36** ***impi**: 86** ***blasphem**: 47**	

